# Comparative efficacy and safety of surgical techniques for inguinal hernia repair in elderly patients: a network meta-analysis

**DOI:** 10.3389/fsurg.2026.1754546

**Published:** 2026-06-11

**Authors:** Xin Yao, Ping Jiang, Jinfu Zheng, Liuzhu Zhang, Jun Wang

**Affiliations:** 1 General Surgery Department, The Second People’s Hospital of Quzhou, Quzhou, Zhejiang, China; 2 Department of Gastroenterology Surgery, The Second Affiliated Hospital, Zhejiang University School of Medicine, Hangzhou, Zhejiang, China

**Keywords:** elderly, inguinal hernia, meta-analysis, safety, surgery

## Abstract

**Objective:**

The optimal surgical technique for elderly patients (≥60 years) with inguinal hernia remains uncertain. This study compared the efficacy and safety of various techniques using network meta-analysis.

**Methods:**

PubMed, Embase, Cochrane Library, and Wanfang databases were searched through February 2025 for randomized controlled trials comparing inguinal hernia repair techniques in elderly patients. Outcomes included perioperative indexes and postoperative complications. Analyses were conducted using R.

**Results:**

Twenty-eight trials (3184 patients; reported mean or median age 62–76 years, predominantly male) were included. The overall risk of bias was judged low in 10 trials, with some concerns in 8 trials and high in 10 trials. The network was well-connected across multiple techniques. Laparoscopic approaches showed advantages in several perioperative outcomes. LR-TEP yielded the least bleeding (vs. OMR-CSR: SMD=−15.18, 95% CI: −23.45 to −6.90) and shortest hospital stay (SMD=−2.30, 95% CI: −3.57 to −1.03), ranking highest for both (SUCRA ≈0.84 and 0.97, respectively). LR-TAPP also reduced bleeding (SMD=−12.34, 95% CI: −24.27 to −0.41) and stay (SMD=−1.81, 95% CI: −3.30 to −0.33). LR-TEP enabled the earliest ambulation (SMD=−18.66, 95% CI: −25.61 to −11.71; SUCRA ≈0.955). Recurrence rates were similar except for OMR-Lightweight, which had a higher risk (RR = 26.16, 95% CI: 1.07–639.67). OMR-PHS had the best safety profile (RR = 0.04, 95% CI: 0.00–0.65). LR-TEP and LR-TAPP significantly reduced complications (RR = 0.28 and 0.33, respectively). IPOM provided the greatest pain relief (SMD=−2.65, 95% CI: −4.58 to −0.73). Urinary retention incidence was low, with no significant between-technique differences.

**Conclusions:**

Minimally invasive repairs, particularly LR-TEP, appear to offer favorable short-term recovery outcomes in appropriately selected elderly inguinal hernia patients, with comparable recurrence rates to open techniques.

**Systematic Review Registration:**

https://www.crd.york.ac.uk/PROSPERO/view/CRD420251124772, identifier PROSPERO 2025 CRD420251124772.

## Introduction

The global population is aging rapidly, and the incidence of groin hernias in older adults is rising in parallel (1). Elderly patients frequently present with inguinal hernias, and approximately 20% have significant comorbidities such as coronary artery disease or chronic pulmonary disease (2). A high comorbidity burden often classifies these patients as high-risk surgical candidates, including those in American Society of Anesthesiologists (ASA) class III or IV (3). Historically, advanced age and frailty have been linked to increased perioperative risk, leading to hesitancy in recommending elective hernia repair in this demographic (4). Concerns have centered on elevated rates of cardiopulmonary complications, wound infections, and perioperative mortality (5). Consequently, surgeons have frequently opted for conservative management or the simplest operative approach—typically open repair under local anesthesia—to minimize physiological stress (6). However, delayed intervention increases the risk of incarceration or strangulation, which carries substantial mortality in elderly patients (7). Determining the safest and most effective surgical strategy for this group therefore remains a clinical challenge.

Over recent decades, inguinal hernia repair techniques have evolved considerably (8, 9). Laparoscopic approaches, including totally extraperitoneal (TEP) and transabdominal preperitoneal (TAPP) repairs, are now widely adopted and offer potential benefits over conventional open Lichtenstein repair, including smaller incisions, less postoperative pain, and faster recovery (10). More recently, robotic-assisted and single-incision laparoscopic techniques have emerged, aiming to further reduce invasiveness and enhance surgical precision (11). These innovations may be particularly advantageous in frail patients, where minimizing surgical trauma is critical. Nonetheless, concerns persist regarding potential risks of laparoscopy in the elderly, including the physiological effects of general anesthesia and pneumoperitoneum on cardiac and pulmonary function (12).

Open surgery has also diversified beyond the traditional Lichtenstein method, with the introduction of techniques such as the Prolene Hernia System, self-gripping meshes, and plug-and-patch repairs, designed to reduce chronic pain or simplify fixation (8, 13). Comparative data on these various open and minimally invasive techniques in elderly patients remain limited, largely confined to small single-center series or subgroup analyses of larger trials (8, 13). While some studies report comparable safety and efficacy of laparoscopic repair in older and younger patients, others suggest higher complication rates or greater technical challenges in elderly individuals, especially in the presence of poorly controlled comorbidities (14, 15).

To date, no comprehensive network meta-analysis has systematically compared the full range of surgical options for inguinal hernia repair in elderly. Network meta-analysis allows integration of direct and indirect evidence, facilitating comparative ranking of multiple interventions even in the absence of head-to-head trials (16). This methodology is particularly valuable given the diversity of available techniques.

Accordingly, we performed a systematic review and network meta-analysis to evaluate the relative efficacy and safety of different inguinal hernia repair methods in elderly patients. We aimed to identify the approaches that optimize short-term recovery (e.g., pain, mobilization, hospital stay) and minimize complications and recurrence, while also delineating potential trade-offs between techniques. These findings are intended to inform surgical decision-making and guideline development for managing inguinal hernias in this growing patient population.

## Methods

### Literature search and selection

This systematic review and network meta-analysis was conducted in accordance with the Preferred Reporting Items for Systematic Reviews and Meta-Analyses (PRISMA) guidelines (17). A comprehensive search was performed to identify studies comparing inguinal hernia repair techniques in elderly patients. We searched PubMed, Embase, the Cochrane Library, and Wanfang databases from inception to February 28, 2025. Search terms included combinations of keywords and MeSH terms related to the patient population (“elderly”, “older adult”), the condition (“inguinal hernia”, “hernia repair”), and surgical techniques (“open repair”, “Lichtenstein”, “laparoscopic”, “TEP”, “TAPP”, “robotic”), as well as outcomes (“complications”, “recurrence”, “mortality”, “pain”, “length of stay”). No language restrictions were applied.

After removing duplicates, two reviewers independently screened titles and abstracts, followed by full-text assessment against the eligibility criteria. Reference lists of included articles and relevant reviews were also hand-searched to identify additional eligible studies.

### Inclusion and exclusion criteria

Studies were eligible if they met the following criteria:

Population: Elderly patients (≥60 years) undergoing inguinal hernia repair, with elderly-specific data available.

Interventions and Comparators: Any surgical technique, including open mesh repair (standard Lichtenstein, heavyweight vs. lightweight mesh, self-gripping mesh, Prolene Hernia System, plug repairs), laparoscopic repair (TAPP or TEP), robotic-assisted repair, or single-incision laparoscopic repair, with at least one comparator.

Outcomes: At least one of the following: postoperative complications, bleeding, pain, time to ambulation, hospital stay, recurrence, wound infection, seroma, urinary retention, or mortality. Of note, seroma was prespecified as a clinically relevant complication, but it was synthesized only if reported separately and consistently across studies.

Study Design: Randomized controlled trials (RCTs) were prioritized; prospective comparative studies were considered if RCT evidence was lacking, although all included studies were ultimately RCTs.

We excluded case series without comparators, studies exclusively involving younger populations without extractable elderly-specific data, and studies in which the risk profile of participants was unclear. For overlapping datasets, the most complete or recent publication was retained.

### Data extraction and quality assessment

Two reviewers independently extracted study and patient characteristics (author, year, country, study setting, sample size, mean/median age, and, when available, age range or proportion of very elderly patients) and details of interventions (technique, surgery type, mesh type). Dichotomous outcomes were extracted as event counts, and continuous outcomes as means with standard deviations. For medians with ranges or interquartile ranges, means and standard deviations were estimated using established methods. Acute complications were recorded at the earliest postoperative time point, and recurrence at the longest reported follow-up. We also attempted to extract surgical site seroma separately from composite wound or local complications. However, seroma was not consistently reported as an independent endpoint in the eligible trials and lack of sufficient data for further analysis.

Risk of bias in RCTs was assessed using the Cochrane Risk of Bias 2.0 tool, evaluating randomization, allocation concealment, blinding, completeness of data, selective reporting, and other biases (18). Discrepancies were resolved by consensus or adjudication by a third reviewer. Since all included studies were RCTs, quality assessment was conducted using tools appropriate for RCTs.

### Data synthesis and statistical analysis

A network meta-analysis using a random-effect model was conducted to integrate direct and indirect evidence and allow for multiple treatment comparisons. For binary outcomes, risk ratios (RRs) with 95% confidence intervals (CIs) were calculated. For continuous outcomes reported on different scales, standardized mean differences (SMDs) with 95% CIs were used. When outcomes were consistently reported in the same units, mean differences were planned but SMDs were ultimately applied for uniformity. Analyses were performed using R 4.4.3 for supplementary diagnostics.

Inconsistency was evaluated globally using the design-by-treatment interaction model, and locally via node-splitting analyses. A *p* < 0.05 indicated significant inconsistency. Loop-specific inconsistency and heterogeneity were also examined. Heterogeneity was quantified using tau^2^ and a global I^2^ statistic.

For treatment ranking, the Surface Under the Cumulative Ranking Curve (SUCRA) was calculated for each intervention and outcome (range 0–1; higher values indicate greater probability of being the best treatment). Publication bias and small-study effects were explored with comparison-adjusted funnel plots and Egger's regression adapted for network meta-analysis (*p* < 0.10 indicating potential asymmetry). All statistical tests were two-sided, with *p* < 0.05 considered statistically significant unless otherwise specified.

## Results

### Study selection and characteristics

**Figure 1:**
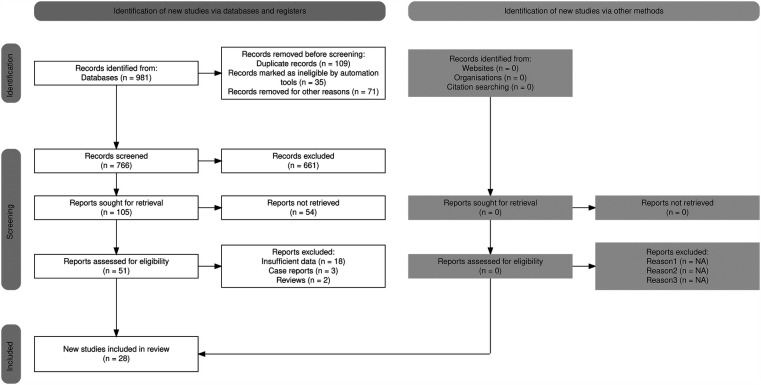
PRISMA flow diagram for study selection. Legend. Flow of records through identification, screening, eligibility, and inclusion up to February 28, 2025; reasons for full-text exclusions are indicated.

The search strategy retrieved 981 records, of which 879 remained after duplicate removal. After title/abstract screening, 51 full-text articles were assessed, and 28 RCTs (8, 19–45) met the eligibility criteria (Figure 1). These trials collectively included 3,184 elderly patients undergoing inguinal hernia repair.

Publications spanned 2016 to 2025, with most conducted in the early 2020s across diverse geographical regions. Trial sample sizes ranged from approximately 50 to 350 patients. Reported mean or median participant age ranged from 62 to 76 years. This range reflects study-level summary statistics rather than the maximum age of all enrolled participants. Men comprised ∼85% of all participants, reflecting the epidemiology of inguinal hernia. By design, all studies enrolled elderly patients (≥60 years). Most procedures were elective; emergency cases were uncommon. The details of included studies are summarized in Table 1.

**Table 1:** Baseline characteristics of included studies.

First author	Year	Sample size (*n*)	Age range (years)	Mean age (years)	Sex (male/female)	Hernia classification (indirect/direct/femoral/recurrent)	Hernia type/stage (I/II/III/IV or as reported)	Laterality	Surgical intervention	Outcomes
Tian Haicheng (29)	2020	54	50–74	62.37 ± 10.39	52/2	42/9/0/3	24/26/4/0	24/26/4/0	Lichtenstein repair	2,3,4,5,7
		53	50–75	61.96 ± 11.12	50/3	41/10/0/2	23/27/3/0	23/27/3/0	Tension-free preperitoneal hernia repair	2,3,4,5,7
Yan Peihu (40)	2022	42	75.2 ± 7.6	75.2 ± 7.6	40/2	32/8/1/1	5/20/16/1	5/20/16/1	Tension-free flat mesh repair (Lichtenstein technique)	2,3,5,6,7
		42	74.6 ± 8.5	74.6 ± 8.5	39/3	33/6/1/2	6/19/16/1	6/19/16/1	Prolene Hernia System repair (PHS)	2,3,5,6,7
		42	76.1 ± 5.7	76.1 ± 5.7	40/2	31/7/2/2	5/18/18/1	5/18/18/1	Mesh plug repair (Rutkow technique)	2,3,5,6,7
Zhang Jiao (30)	2020	50	62-80	69.26 ± 6.55	50/0				Tension-free hernia repair (Lichtenstein)	2,3,4,5,7
		50	61–79	70.53 ± 6.35	50/0				Laparoscopic transabdominal preperitoneal inguinal hernia mesh repair (TAPP)	2,3,4,5,7
Qu Feng (38)	2022	40	60–85	67.31 ± 3.16	32/8	28/12/0/0	28/12/0/0		Laparoscopic totally extraperitoneal hernia repair (TEP)	2,3,4,5,6,7
		40	61–83	68.29 ± 2.98	33/7	27/13/0/0	27/13/0/0		Laparoscopic transabdominal preperitoneal hernia repair (TAPP)	2,3,4,5,6,7
Su Jinkun (34)	2021	25	60–89	73.18 ± 5.19	13/12	12/13/0/0	12/13/0/0		Transabdominal preperitoneal hernia repair (TAPP)	1,2,3,4,5,7
		25	61–88	72.56 ± 5.74	14/11	10/15/0/0	10/15/0/0		Plug-based tension-free inguinal hernia repair	1,2,3,4,5,7
Dong Kai (43)	2023	30	60–81	68.45 ± 6.32	26/4				Conventional hernia repair	2,3,5,7
		30	60–79	68.37 ± 6.45	25/5				Extraperitoneal laparoscopic hernia repair (TEP)	2,3,5,7
Liu Weidong (25)	2018	40	66.4 ± 4.8	66.4 ± 4.8	34/6			unilateral 36 cases, bilateral 4 cases	Conventional hernia repair	2,3,4,5,7
		40	66.8 ± 5.2	66.8 ± 5.2	36/4			unilateral 35 cases, bilateral 5 cases	Extraperitoneal laparoscopic hernia repair (TEP)	2,3,4,5,7
Hou Haisheng (27)	2020	80	45.6 ± 14.8	45.6 ± 14.8	73/7			unilateral hernia	Laparoscopic TAPP mesh implantation using a lightweight 3D Max mesh without fixation	7
		62	46.3 ± 13.7	46.3 ± 13.7	58/4			unilateral hernia	Laparoscopic TAPP mesh implantation using a standard polypropylene mesh fixed with tacks	7
Wang Lishuang (22)	2017	175	71–76	73.4 ± 2.03	132/43				Laparoscopic transabdominal preperitoneal hernia repair (TAPP)	3,7
		175	70–75	72.8 ± 1.67	143/32				Laparoscopic totally extraperitoneal hernia repair (TEP)	3,7
Zhu Bailian (41)	2022	42	70.24 ± 6.89	70.24 ± 6.89	37/5	29/7/0/6		unilateral hernia 32 cases, bilateral hernia 10 cases	Laparoscopic totally extraperitoneal hernia repair (TEP)	2,3,4,5,6,7
		42	69.82 ± 6.97	69.82 ± 6.97	38/4	31/8/0/3		unilateral hernia 31 cases, bilateral hernia 11 cases	Tension-free hernia repair	2,3,4,5,6,7
Duan Chao (32)	2021	35	58.83 ± 3.58	58.83 ± 3.58	31/4			right side 20 cases, left side 15 cases	Laparoscopic hernia repair	2,3,5,6,7
		35	58.29 ± 5.75	58.29 ± 5.75	30/5			right side 21 cases, left side 14 cases	Open posterior inguinal canal wall repair	2,3,5,6,7
Fan Ling (36)	2022	52	69.76 ± 4.75	69.76 ± 4.75	45/7	42/18/3/2	42/18/3/2		Laparoscopic totally extraperitoneal hernia repair (TEP)	3,4,5,6,7
		47	69.12 ± 4.05	69.12 ± 4.05	42/5	47/14/2/1	47/14/2/1		Laparoscopic transabdominal preperitoneal hernia repair (TAPP)	3,4,5,6,7
Liang Jiangchun (24)	2018	50	67.3 ± 5.6	67.3 ± 5.6	42/18				Laparoscopic hernia repair	2,3,4,5,7
		50	66.4 ± 6.8	66.4 ± 6.8	41/19				Mesh plug repair	2,3,4,5,7
Zhou Zhijian (31)	2020	30	61–87	67.23 ± 2.48	16/14				Preperitoneal hernia repair	6,7
		30	63–88	67.46 ± 2.74	17/13				Laparoscopic intraperitoneal onlay mesh repair (IPOM)	6,7
Fan Liping (44)	2023	63	68.78 ± 2.22	68.78 ± 2.22	45/18				Mesh plug repair	1,2,3,4,5,7
		63	68.41 ± 2.81	68.41 ± 2.81	40/23				Laparoscopic repair	1,2,3,4,5,7
Pan Honggang (20)	2016	40	60–79	66.8 ± 1.7	38/2	9/29/2/0		unilateral hernia 32 cases, bilateral hernia 8 cases	Conventional open tension-free hernia repair	3,5,7
		40	60–77	67.9 ± 3.2	36/4	12/27/1/0	12/27/1/0	unilateral hernia 34 cases, bilateral hernia6 cases	Minimally invasive laparoscopic tension-free hernia repair	3,5,7
Liu Yonggang (26)	2019	148	61–79	69.0 ± 4.3	81/67	direct hernia 52 cases, indirect hernia 96 cases	direct hernia 52 cases, indirect hernia 96 cases	direct hernia 52 cases, indirect hernia 96 cases	Millikan treatment (open tension-free inguinal hernia repair)	2,3,4,6,7
		150	60–78	69.3 ± 4.5	81/69	direct hernia 43 cases, indirect hernia 107 cases	direct hernia 43 cases, indirect hernia 107 cases	direct hernia 43 cases, indirect hernia 107 cases	TEP treatment (laparoscopic totally extraperitoneal inguinal hernia repair)	2,3,4,6,7
Huang Shangyu (28)	2020	30	55–75	65.91 ± 4.38	26/4				Open tension-free hernia repair	1,2,3,4,5,7
		30	55–75	65.39 ± 4.15	25/5				Laparoscopic inguinal hernia repair	1,2,3,4,5,7
Zhu Kui (23)	2018	47	62–87	67.8 ± 2.8	43/4	12/33/2/0	0/25/19/3	unilateral hernia 35 cases, bilateral hernia12 cases	Laparoscopic totally extraperitoneal inguinal hernia repair (TEP)	3,5,6,7
		47	60–87	67.5 ± 2.7	44/3	11/33/3/0	0/24/20/3	unilateral hernia 36 cases, bilateral hernia11 cases	Open plug-based tension-free inguinal hernia repair	3,5,6,7
Ruan Zhaojie (39)	2022	40	≥70	75.4 ± 5.3	37/3	28/12/0/0	28/12/0/0		Totally extraperitoneal hernia repair (TEP)	2,3,6,7
		40	≥70	74.7 ± 6.2	39/1	31/9/0/0			Transabdominal preperitoneal hernia repair (TAPP)	2,3,6,7
Yang Lizhi (21)	2016	50	46–78	58.50 ± 7.90	41/9				Tension-free hernia repair with lightweight mesh	4,5
		50	45–76	58.45 ± 7.82	40/10				Tension-free hernia repair with heavyweight mesh	4,5
Yu Luyao (35)	2021	35	67.8 ± 2.2	67.8 ± 2.2	17/18	direct hernia 12 cases, indirect hernia 17 cases, bilateral hernia 6 cases	direct hernia 12 cases, indirect hernia 17 cases, bilateral hernia 6 cases	direct hernia 12 cases, indirect hernia 17 cases, bilateral hernia 6 cases	Tension-free hernia repair plus standard mesh	2,3,5,7
		35	68.2 ± 2.4	68.2 ± 2.4	18/17	direct hernia 11 cases, indirect hernia 19 cases, bilateral hernia 5 cases	direct hernia 11 cases, indirect hernia 19 cases, bilateral hernia 5 cases	direct hernia 11 cases, indirect hernia 19 cases, bilateral hernia 5 cases	Tension-free hernia repair plus lightweight mesh	2,3,5,7
Liu Xing (45)	2025	36	72.53 ± 8.58	72.53 ± 8.58	36/0			unilateral hernia	Totally extraperitoneal hernia repair (TEP)	2,3,4,5,6,7
		65	74.31 ± 9.52	74.31 ± 9.52	65/0			unilateral hernia	Preperitoneal hernia repair	2,3,4,5,6,7
Feng Xueshu (37)	2022	50	69.57 ± 4.35	69.57 ± 4.35	46/4	33/17/0/0	33/17/0/0		Mesh plug repair	3,4,5,6,7
		50	70.12 ± 4.17	70.12 ± 4.17	44/6	30/20/0/0	30/20/0/0		Lichtenstein repair	3,4,5,6,7
		50	71.38 ± 3.57	71.38 ± 3.57	47/3	31/19/0/0	31/19/0/0		Laparoscopic transabdominal preperitoneal repair (TAPP)	3,4,5,6,7
Huang Cuijing (33)	2021	41	61–86	68.2 ± 6.5	33/8		3/16/18/4	unilateral hernia	Laparoscopic totally extraperitoneal hernia repair (TEP)	2,3,4,5,7
		37	62–84	69.3 ± 7.1	26/11		2/13/19/3	unilateral hernia	Lichtenstein tension-free hernia repair under local anesthesia	2,3,4,5,7
Alessia Ferrarese (19)	2016	30			30/0	indirect hernia: 27 (90%), direct hernia: 3 (10%)	primary: 28 (86.6%), recurrent: 4 (13.3%)	primary: 28 (86.6%), recurrent: 4 (13.3%)	Laparoscopic TAPP with polypropylene mesh fixed by fibrin glue	3,5,6,7
		30			30/0	indirect hernia: 26 (86.6%), direct hernia: 4 (13.3%)	primary: 25 (83.3%), recurrent: 5 (16.6%)	primary: 25 (83.3%), recurrent: 5 (16.6%)	Laparoscopic TAPP with self-fixating mesh	3,5,6,7
M. Ceresoli (42)	2020					inguinal hernia: 10 cases(100%)	inguinal hernia: 10 cases(100%)	inguinal hernia: 10 cases(100%)	Laparoscopic repair	
						inguinal hernia: 170 cases(75.22%), femoral hernia: 79 cases(24.78%)	inguinal hernia: 170 cases(75.22%), femoral hernia: 79 cases(24.78%)	inguinal hernia: 170 cases(75.22%), femoral hernia: 79 cases(24.78%)	Open repair (direct repair or mesh repair)	
Mehmet Esref Ulutas (8)	2025	60	71.7 ± 6.5	71.7 ± 6.5	60/0	indirect hernia: 37 (61.7%), direct hernia: 18 (30%), femoral hernia: 2 (3.3%), lipoma: 3 (5%)	indirect hernia: 37 (61.7%), direct hernia: 18 (30%), femoral hernia: 2 (3.3%), lipoma: 3 (5%)	right side: 34 (56.7%), left side: 26 (43.3%)	Open surgery (Lichtenstein)	3,4,5,6,7
		60	69.6 ± 3.9	69.6 ± 3.9	60/0	indirect hernia: 37 (61.7%), direct hernia: 17 (28.3%), femoral hernia: 2 (3.3%), lipoma: 4 (6.7%)	indirect hernia: 37 (61.7%), direct hernia: 17 (28.3%), femoral hernia: 2 (3.3%), lipoma: 4 (6.7%)	right side: 36 (60%), left side: 24 (40%)	Laparoscopic totally extraperitoneal repair (TEP)	3,4,5,6,7

Outcome codes: 1, at least one efficacy-related endpoint (overall response/effective response/excellent or good therapeutic effect); 2, intraoperative blood loss (mL); 3, operation time (min); 4, time to ambulation (h); 5, length of hospital stay (days); 6, at least one VAS score before or after surgery at different time points; 7, at least one complication-related endpoint. NR, not reported; TAPP, transabdominal preperitoneal repair; TEP, totally extraperitoneal repair; PHS, Prolene Hernia System; IPOM, intraperitoneal onlay mesh.

### Risk of bias

**Figure 2:**
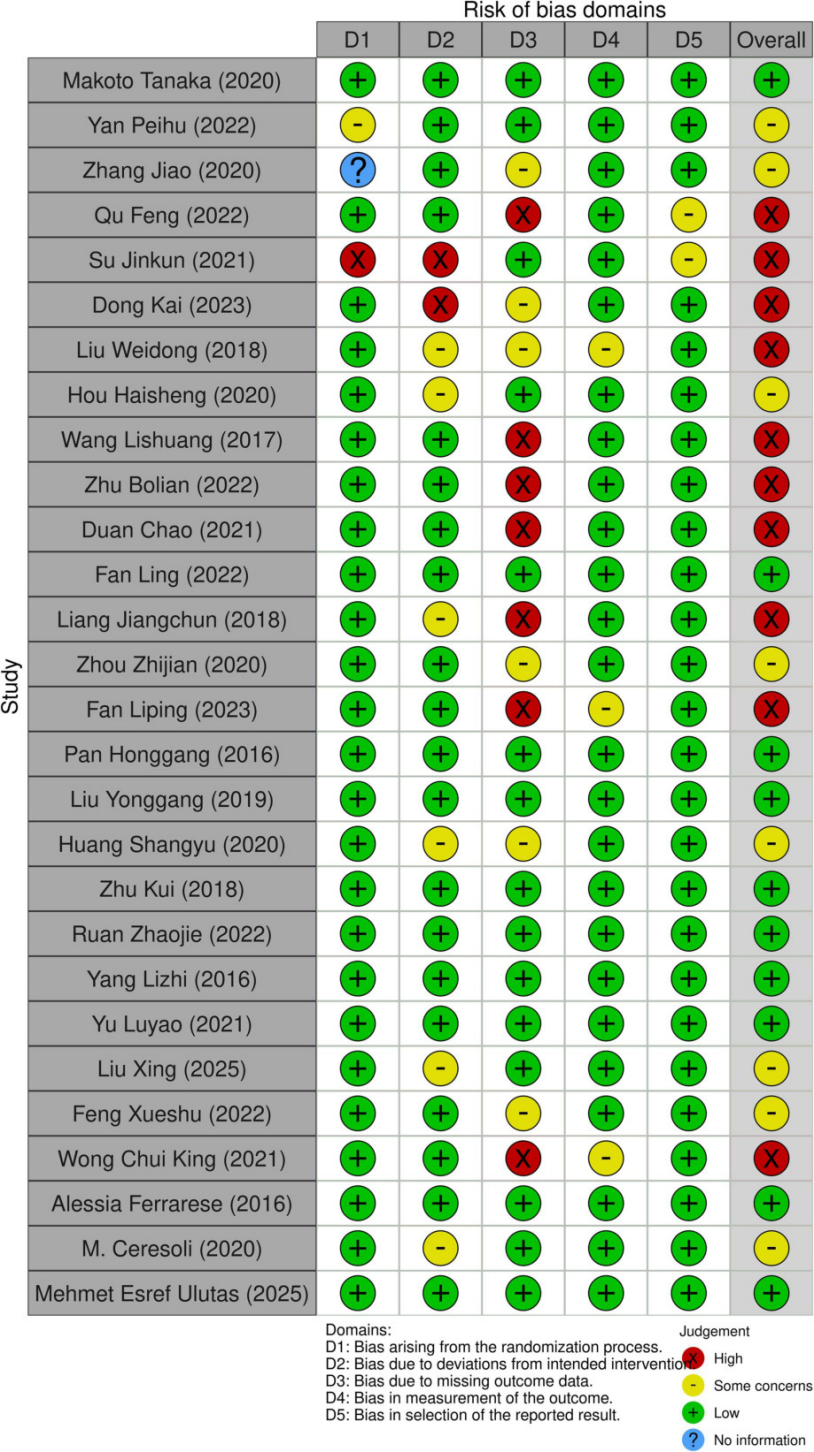
Risk of bias (RoB 2) assessment across included randomized controlled trials. Legend. Risk of bias judgments for 28 RCTs using the RoB 2 tool. Overall risk of bias: Low 10 (35.7%), Some concerns 8 (28.6%), High 10 (35.7%). Domain-level results: D1—Randomization process: Low 25 (89.3%), Some concerns 2 (7.1%), High 1 (3.6%). D2—Deviations from intended interventions: Low 20 (71.4%), Some concerns 6 (21.4%), High 2 (7.1%). D3—Missing outcome data: Low 15 (53.6%), Some concerns 6 (21.4%), High 7 (25.0%). D4—Measurement of the outcome: Low 25 (89.3%), Some concerns 3 (10.7%). D5—Selection of the reported result: Low 26 (92.9%), Some concerns 2 (7.1%). Inherent challenges with blinding in surgical trials may influence subjective outcomes; several studies mitigated risk via blinded outcome assessment and focus on objective endpoints.

Based on the RoB 2 assessment of the 28 included RCTs (8, 19–45), the overall risk of bias was judged low in 10 (35.7%), some concerns in 8 (28.6%), and high in 10 (35.7%). Domain-level judgments were as follows: D1—randomization process: low 25 (89.3%), some concerns 2 (7.1%), high 1 (3.6%); D2—deviations from intended interventions: low 20 (71.4%), some concerns 6 (21.4%), high 2 (7.1%); D3—missing outcome data: low 15 (53.6%), some concerns 6 (21.4%), high 7 (25.0%); D4—measurement of the outcome: low 25 (89.3%), some concerns 3 (10.7%); D5—selection of the reported result: low 26 (92.9%), some concerns 2 (7.1%). Recognizing the practical challenges of blinding in surgical trials, several studies mitigated detection bias through blinded outcome assessment or reliance on objective endpoints. Sensitivity analyses excluding studies at high risk of bias yielded results consistent with the primary findings (Figure 2).

### Interventions and network structure

**Figure 3:**
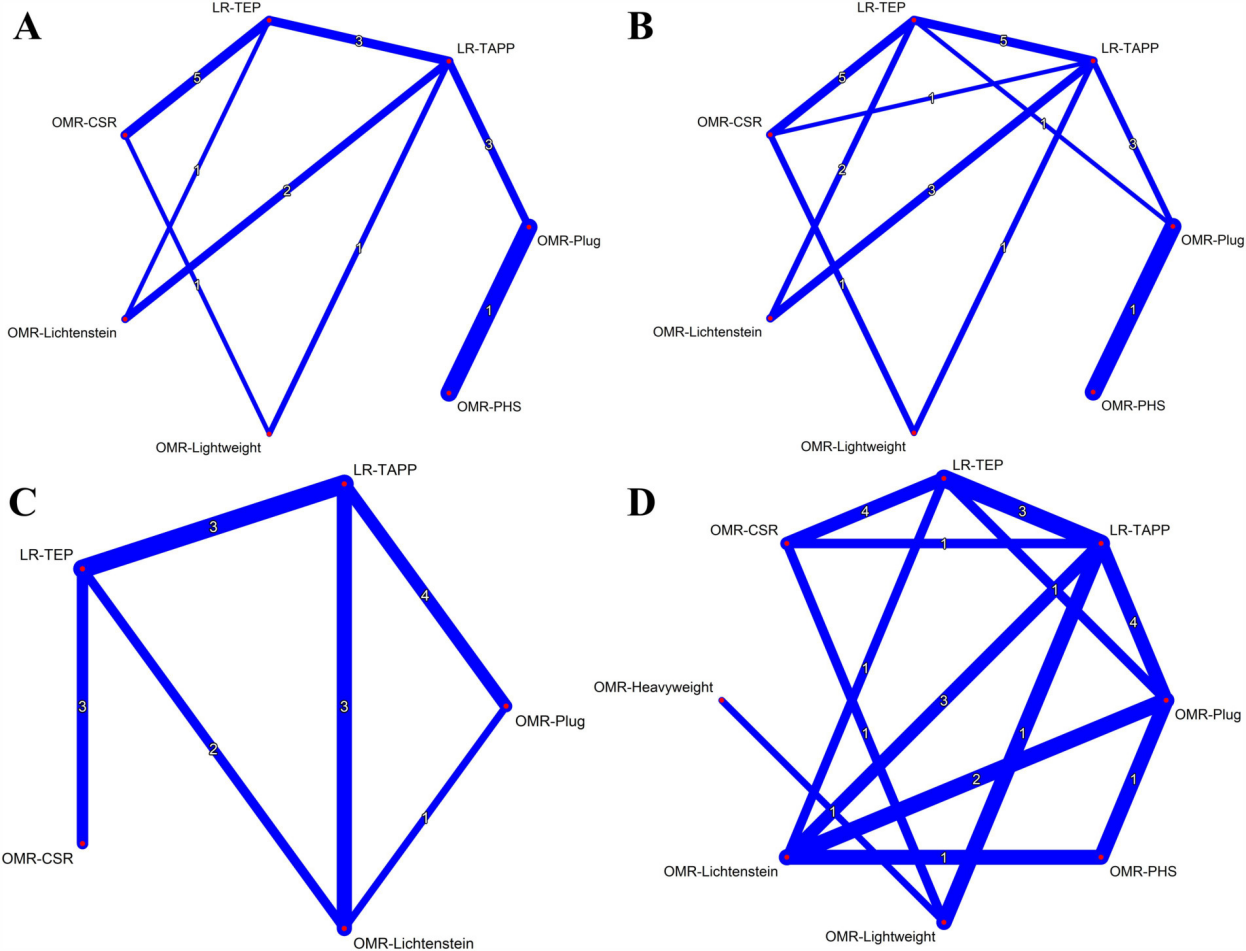
Network geometry by outcome. **(A)** Bleeding; **(B)** Operation duration; **(C)** Time to ambulation (off-bed time); **(D)** Hospital stay. Nodes represent interventions (LR-TEP, LR-TAPP, OMR-Lichtenstein, OMR-PHS, OMR-Plug, OMR-Heavyweight, OMR-Lightweight, OMR-CSR, IPOM); node size reflects total sample size and edge thickness the number of direct comparisons. The network is well connected, with LR-TEP and LR-TAPP central to most contrasts; some newer techniques (e.g., OMR-CSR) have limited direct evidence.

**Figure 4:**
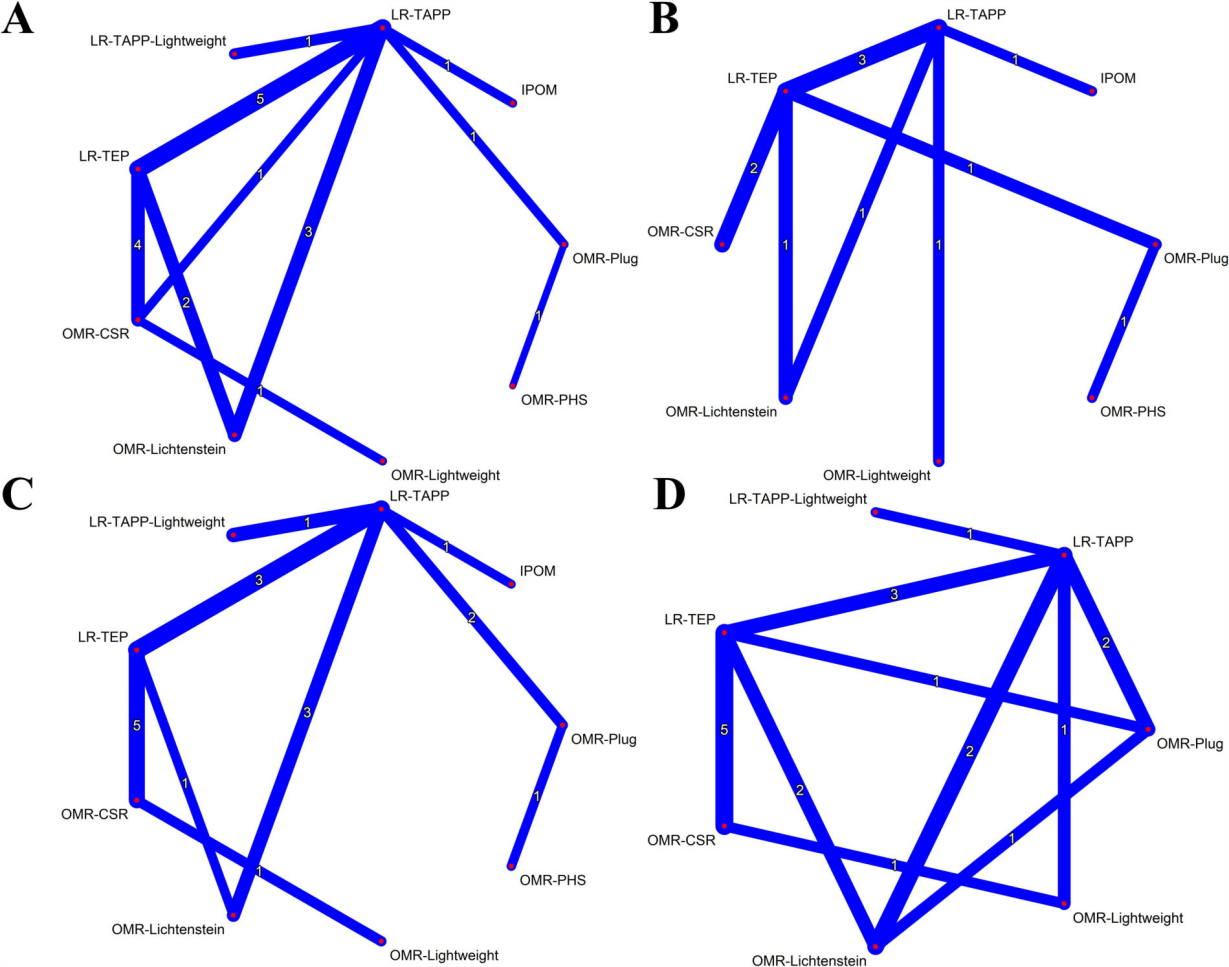
Network geometry by safety. **(A)** Postoperative pain; **(B)** Any postoperative complication (overall safety); **(C)** Urinary retention; **(D)** Wound infection. As in Figure 3; network geometry is shown for safety outcomes. LR-TEP and LR-TAPP remain central nodes, whereas several open-mesh variants contribute fewer direct comparisons.

The network included both laparoscopic and open repair techniques (Figures 3, 4):

Laparoscopic: LR-TEP (totally extraperitoneal), LR-TAPP (transabdominal preperitoneal), and one robotic-assisted TEP (pooled with LR-TEP).

Open: OMR-Lichtenstein, OMR-Heavyweight, OMR-Lightweight, OMR-PHS (Prolene Hernia System), OMR-Plug, and OMR-CSR (composite self-retaining mesh).

Other: IPOM (intraperitoneal onlay mesh).

The network geometry (Figures 3, 4) demonstrated robust connectivity. Laparoscopic vs. open repairs formed the most common direct comparisons (e.g., LR-TAPP vs. OMR-Plug, LR-TEP vs. LR-TAPP). OMR-Lichtenstein and OMR-CSR were frequent control arms.

### Perioperative outcomes

#### Postoperative bleeding

**Figure 5:**
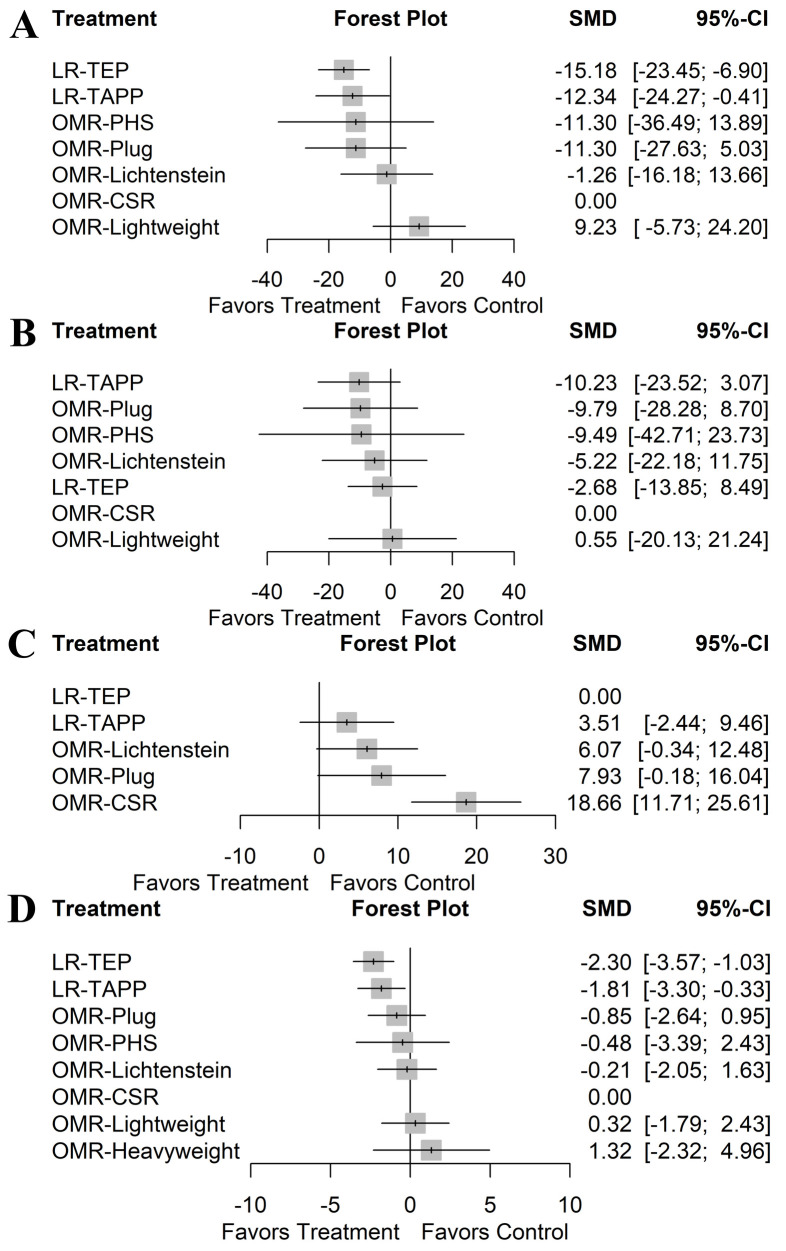
Network forest plots for primary outcomes. **(A)** Bleeding; **(B)** Operation duration; **(C)** Time to ambulation (off-bed time); **(D)** Hospital stay. Network treatment effects versus the prespecified reference (e.g., OMR-CSR) with 95% CIs. Standardized mean differences (SMDs) are reported for continuous outcomes; SMD < 0 favors less bleeding, shorter operative time, earlier ambulation, or shorter length of stay as appropriate. The vertical line denotes no effect.

Seven interventions were evaluated (Figure 5A). LR-TEP achieved the largest bleeding reduction compared with OMR-CSR (SMD=−15.18; 95% CI, −23.45 to −6.90), followed by LR-TAPP (SMD=−12.34; 95% CI, −24.27 to −0.41). No significant differences were observed between open techniques. OMR-Lightweight trended toward greater bleeding (SMD=9.23; 95% CI, −5.73 to 24.20). SUCRA: LR-TEP (0.84), LR-TAPP (0.70) highest; OMR-Lightweight lowest (0.10) (Supplementary Figure 1).

#### Operation duration

Eight interventions were assessed (Figure 5B). LR-TAPP ranked highest for shortest operative time (SUCRA=0.754), followed by OMR-Plug (0.690) and OMR-PHS (0.608). LR-TAPP showed the most favorable point estimate (SMD=−10.23; 95% CI, −23.52 to 3.07). OMR-Plug (SMD=−9.79; 95% CI, −28.28 to 8.70) and OMR-PHS (SMD=−9.49; 95% CI, −42.71 to 23.73) demonstrated similar trends. OMR-Lichtenstein had a moderate SMD (–5.22; 95% CI, −22.18 to 11.75). LR-TEP (SMD=−2.68; 95% CI, −13.85 to 8.49), OMR-CSR (0.00; 95% CI, −13.06 to 13.06), and OMR-Lightweight (0.55; 95% CI, −20.13 to 21.24) were associated with longer durations. These findings suggest that LR-TAPP and certain open mesh variants (OMR-Plug, OMR-PHS) may reduce anesthesia exposure—an important consideration in elderly population (Supplementary Figure 2).

#### Time to ambulation

Five interventions were analyzed (Figure 5C). LR-TEP facilitated the earliest mobilization (SMD=18.66; 95% CI, 11.71 to 25.61 vs. OMR-CSR). LR-TAPP trended toward faster ambulation (SMD=3.51; 95% CI, −2.44 to 9.46). SUCRA: LR-TEP (0.955) highest, OMR-CSR lowest (Supplementary Figure 3).

#### Length of hospital stay

Ten interventions were included (Figure 5D). LR-TEP (SMD=−2.30; 95% CI, −3.57 to −1.03) and LR-TAPP (SMD=−1.81; 95% CI, −3.30 to −0.33) significantly shortened stays compared with OMR-CSR. No differences were detected between open variants. SUCRA values were highest for LR-TEP (0.97) and LR-TAPP (0.85) (Supplementary Figure 4). Absolute length of stay in the trials should be interpreted cautiously because discharge policies varied by country, hospital pathway, year of recruitment, and patient risk profile.

### Long-term efficacy: hernia recurrence

**Figure 6:**
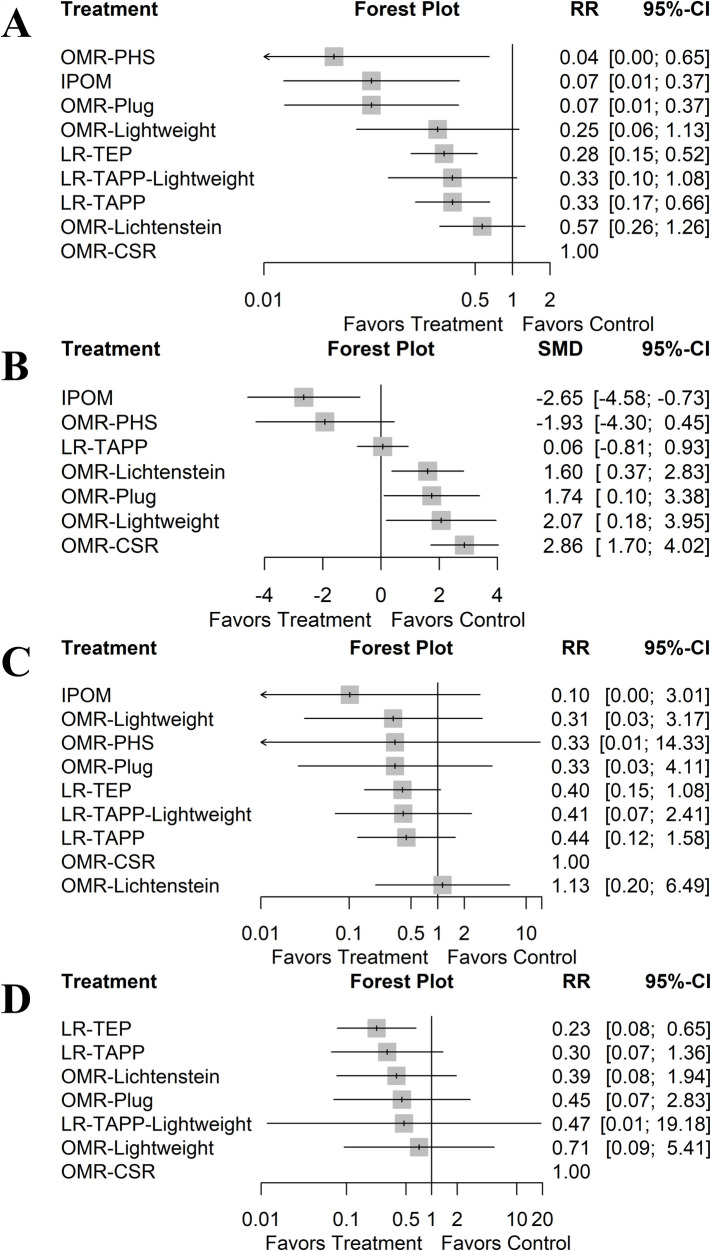
Network forest plots for safety outcomes. **(A)** Any postoperative complication (overall safety); **(B)** Postoperative pain; **(C)** Urinary retention; **(D)** Wound infection. Network treatment effects versus the prespecified reference (e.g., OMR-CSR) with 95% CIs. Risk ratios (RRs) are used for dichotomous outcomes (RR < 1 favors the treatment); SMDs are used for pain (SMD < 0 favors less pain).

Nine interventions were analyzed (Figure 6A). Recurrence rates were low and generally similar across techniques (Figure 6B). OMR-Lightweight had a significantly higher recurrence risk than OMR-CSR (RR = 26.16; 95% CI, 1.07 to 639.67). SUCRA ranked OMR-CSR highest (0.81), OMR-Lightweight lowest (Supplementary Figure 5).

### Safety outcomes

#### Composite complication rate

**Figure 7:**
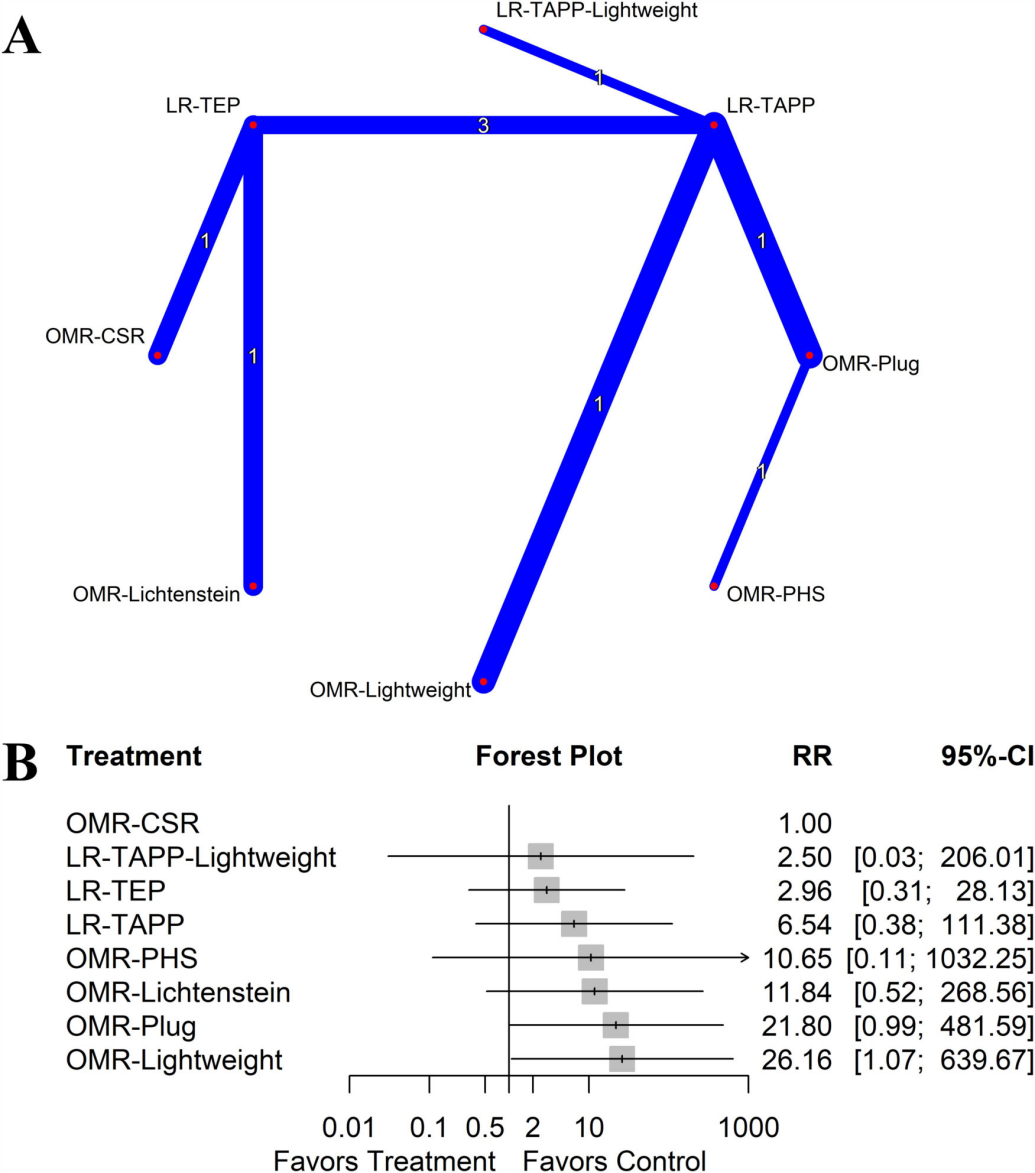
Network meta-analysis for recurrence. **(A)** Network geometry for recurrence; **(B)** Network forest plot showing RRs with 95% CIs versus the reference (RR < 1 favors the treatment). Node size and edge thickness as in Figure 3; the forest plot summarizes network estimates for hernia recurrence.

Ten interventions were evaluated (Figure 7A). OMR-PHS had the most favorable safety profile (RR = 0.04; 95% CI, 0.00 to 0.65 vs. OMR-CSR). IPOM and OMR-Plug each showed RR = 0.07 (95% CI, 0.01 to 0.37). LR-TEP (RR = 0.28; 95% CI, 0.15 to 0.52) and LR-TAPP (RR = 0.33; 95% CI, 0.17 to 0.66) reduced complication risk. SUCRA: OMR-PHS highest, followed by IPOM, OMR-Plug, LR-TEP, and LR-TAPP (Supplementary Figure 6).

#### Postoperative pain

Ten interventions were analyzed (Figure 7B). IPOM produced the greatest pain reduction (SMD=−2.65; 95% CI, −4.58 to −0.73 vs. OMR-CSR). OMR-CSR (SMD=2.86; 95% CI, 1.70 to 4.02) and OMR-Lightweight (SMD=2.07; 95% CI, 0.18 to 3.95) were associated with higher pain. SUCRA: IPOM (0.95) highest, OMR-CSR lowest (Supplementary Figure 7).

#### Urinary retention

Nine interventions were assessed (Figure 7C). No statistically significant differences were detected; point estimates favored IPOM, OMR-Lightweight, and OMR-Plug, but all CIs were wide. The SUCRA ranking is presented in Supplementary Figure 8.

#### Wound infection

Although event counts were low, network estimates could be obtained using OMR-CSR as the reference (Figure 7D). LR-TEP was associated with a significantly lower risk of wound infection (RR = 0.23, 95% CI 0.08–0.65). Directionally lower but not statistically significant reductions were observed with LR-TAPP (RR = 0.30, 95% CI 0.07–1.36), OMR-Lichtenstein (RR = 0.39, 95% CI 0.08–1.94), OMR-Plug (RR = 0.45, 95% CI 0.07–2.83), LR-TAPP with lightweight mesh (RR = 0.47, 95% CI 0.01–19.18), and OMR-Lightweight (RR = 0.71, 95% CI 0.09–5.41). Confidence intervals were wide for several techniques, reflecting sparse events, but no technique showed an increased risk relative to OMR-CSR. Perioperative mortality remained rare (<1% across studies) with no significant differences between interventions. For SUCRA ranking, consistent with the forest-plot estimates, the SUCRA values for wound infection ranked the interventions (Supplementary Figure 9) as: LR-TEP (0.802), LR-TAPP (0.681), OMR-Lichtenstein (0.540), LR-TAPP–Lightweight (0.486), OMR-Plug (0.479), OMR-Lightweight (0.328), and OMR-CSR (0.184). Surgical site seroma was reviewed but was not reported consistently as a distinct outcome, and consequently no separate network estimate or ranking was calculated for seroma.

**Figure 8:**
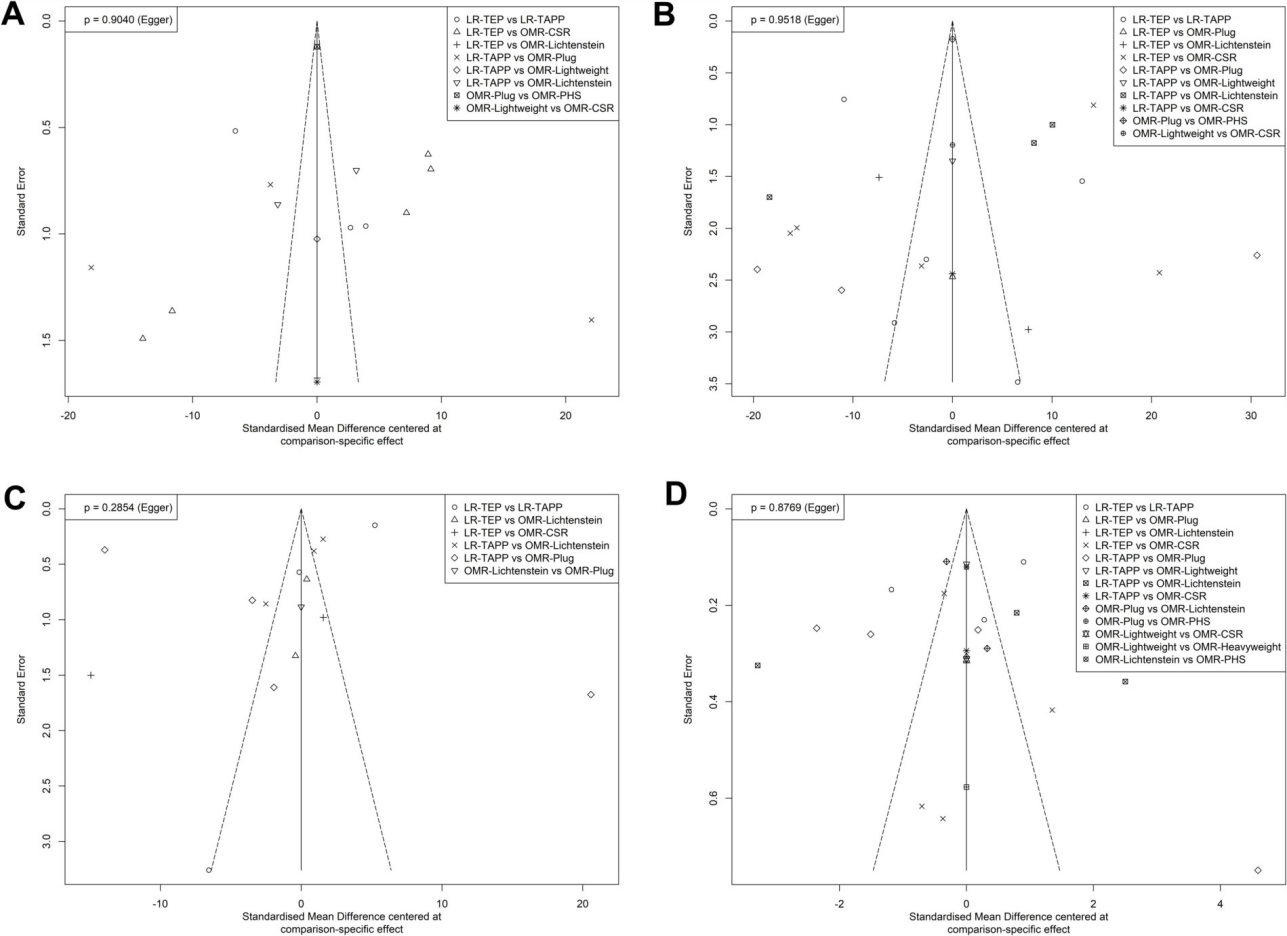
Funnel plots for primary outcomes. **(A)** Bleeding; **(B)** Operation duration; **(C)** Time to ambulation (off-bed time); **(D)** Hospital stay. Comparison-adjusted funnel plots centered at the comparison-specific effect; dashed lines indicate pseudo-95% limits. Plots appear symmetric; for operation duration, Egger's test *p* = 0.9518 indicates no evidence of small-study effects.

**Figure 9:**
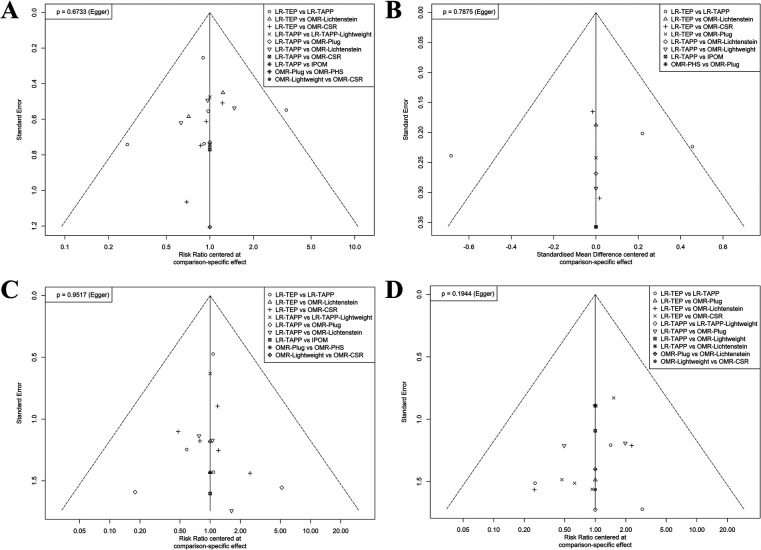
Funnel plots for safety outcomes. **(A)** Any postoperative complication (overall safety); **(B)** Postoperative pain; **(C)** Urinary retention; **(D)** Wound infection. Comparison-adjusted funnel plots as in Figure 8. No systematic asymmetry was observed, suggesting an absence of small-study effects/publication bias for these outcomes.

Funnel plots for bleeding, hospital stay, pain, and operation duration were symmetrical (Figures 8, 9). Egger's tests were non-significant (e.g., operation duration *p* = 0.9518), suggesting no evidence of publication bias or small-study effects.

#### Heterogeneity, and network validity

Heterogeneity varied by outcome (Supplementary Figures 10, 17). Bleeding and pain showed high between-study variability (I^2^ approaching 99%), while operation duration demonstrated substantial heterogeneity for LR-TAPP vs. OMR-CSR and LR-TAPP vs. OMR-Lightweight. In contrast, comparisons involving OMR-Plug (e.g., LR-TEP vs. OMR-Plug) showed low heterogeneity.

Contribution analysis indicated that some operation duration estimates relied heavily on indirect evidence (e.g., LR-TAPP vs. OMR-CSR with 23.7% direct evidence), whereas others (e.g., OMR-PHS vs. OMR-Plug) were entirely supported by direct data.

## Discussion

This network meta-analysis provides a comprehensive comparison of inguinal hernia repair techniques in elderly patients, an area that remains incompletely addressed in the existing literature. The findings demonstrate that surgical technique influences outcomes in this vulnerable population, with several clinically important insights emerging.

For short-term perioperative outcomes and overall safety, minimally invasive techniques, particularly laparoscopic approaches, demonstrated clear advantages over conventional open surgery (8, 46). LR-TEP consistently ranked among the top interventions, associated with the least blood loss, shortest hospital stay, and fastest postoperative mobilization (46–48). LR-TAPP also outperformed open techniques across many parameters (10, 49), although effect sizes were generally smaller than those for LR-TEP. These advantages are clinically meaningful, as even modest reductions in hospitalization or immobility can reduce the risk of complications such as thromboembolism, muscle deconditioning, and nosocomial infection in elderly patients. Reduced surgical trauma and postoperative pain with laparoscopy likely facilitate earlier ambulation and discharge. Importantly, recurrence rates with laparoscopic techniques were not statistically different from those observed with open mesh repair, addressing concerns that laparoscopy might compromise repair durability in older patients.

Certain modern open repair techniques also demonstrated strengths. The Prolene Hernia System repair exhibited the lowest complication rates and very low pain scores, suggesting that its bilayer design and minimal fixation requirements may reduce tissue trauma and postoperative morbidity. Similarly, mesh plug repair achieved favorable safety profiles, consistent with earlier reports of its tolerability in older patients (50). In contrast, open lightweight mesh repairs were associated with a significantly higher recurrence risk without a clear reduction in pain. This suggests that, in elderly patients, the theoretical benefits of lightweight mesh may be outweighed by reduced mechanical durability.

These findings support wider consideration of laparoscopic repair in elderly patients who can tolerate general anesthesia, provided the surgeon and center have adequate experience with the selected technique. Advances in anesthesia, such as short-acting agents and meticulous perioperative monitoring, have improved the safety of general anesthesia even in ASA III patients. In such cases, the benefits of reduced pain, earlier mobilization, and fewer complications may outweigh the anesthetic risks (51, 52). However, the generalizability of these benefits depends on the learning curve, operative volume, case selection, and perioperative pathway of each center.

For patients unsuitable for laparoscopy, our results highlight the value of tailored open approaches. Techniques such as PHS or plug-and-patch repair may offer superior outcomes compared to standard Lichtenstein repair, particularly in minimizing postoperative pain and complications (53, 54). These techniques also avoid pneumoperitoneum, which may be advantageous in patients with severe cardiopulmonary disease. However, caution is warranted with lightweight mesh in open repairs, as the recurrence risk appears elevated in this population.

Irrespective of technique, multidisciplinary preoperative optimization remains critical. Many complications in elderly patients arise from comorbid conditions rather than the surgical site. Preoperative cardiopulmonary assessment, optimization of chronic conditions (including diabetes, COPD, heart failure, and chronic renal impairment), medication review, and targeted interventions such as pulmonary physiotherapy can help mitigate these risks (55, 56). For frail patients, preoperative exercise and conditioning programs may improve functional reserve and should be considered when the clinical situation allows (57). Early mobilization, adequate analgesia, and enhanced recovery protocols adapted for hernia surgery may further improve outcomes.

Although our analysis focused on elective repairs, background evidence indicates that emergency hernia surgery in elderly patients carries substantially higher morbidity and mortality (58, 59). These data support a proactive approach to repairing symptomatic or high-risk hernias electively once comorbidities are optimized.

The present findings are consistent with prior meta-analyses in mixed-age populations showing that laparoscopic repair reduces acute pain and wound complications compared with open repair, without compromising recurrence rates (59–61). Our results extend these observations to elderly cohorts. Köckerling et al. (62) previously reported the feasibility and safety of TEP repair in octogenarians, which our analysis corroborates in a broader set of laparoscopic and open techniques.

Some observational studies have noted higher complication rates for laparoscopic repair in elderly vs. younger patients (63). While we did not directly compare age groups, our results demonstrate that within the elderly population, laparoscopic repair yields fewer complications than open repair, which is clinically relevant for surgical decision-making. The favorable outcomes associated with PHS in our analysis may reflect its particular suitability in elderly patients, as the reduced fixation and bilayer design may confer advantages in frail tissues.

Several limitations should be acknowledged. First, the included RCTs often had small sample sizes and limited follow-up, precluding assessment of rare outcomes such as chronic pain syndromes or very late recurrences. Second, there was clinical heterogeneity: “elderly” was variably defined, and the degree of surgical risk differed across trials. The reported age range primarily reflects study-level mean or median ages, and patients aged 80 years or older were not consistently reported. As such, our findings represent average treatment effects across a broad range of patient profiles and should be interpreted in the context of individual patient factors. Third, some interventions, such as composite self-retaining mesh or robotic repair, were represented by only one or two trials, making their estimates less precise.

We also encountered incomplete reporting for certain outcomes, particularly surgical site seroma, wound infection, discharge criteria, and long-term operative outcomes. Seroma is a common postoperative issue after laparoscopic repair, but it was usually embedded within composite local complications or omitted; therefore, we could not perform a separate network analysis. Although laparoscopic repair is generally associated with lower wound infection rates, sparse event counts limited precision. Operative duration, an important consideration in anesthesia risk for elderly patients, was variably reported; our available data suggest that LR-TAPP and some open mesh techniques (e.g., OMR-Plug, OMR-PHS) may offer time efficiency advantages over others, but further research is warranted. Differences in hospital stay should also be interpreted in light of center-specific discharge policy, local reimbursement systems, enhanced recovery implementation, and whether ambulatory or 24-hour discharge pathways were routinely used.

Minor inconsistencies were observed between direct and indirect estimates for a small number of comparisons, likely reflecting single-trial outliers, differences in patient selection, center-level practice patterns, or variation in surgeon expertise. Some centers may preferentially adopt and refine one procedure, and outcomes achieved in high-volume specialist settings may not be fully reproducible in lower-volume centers. Although these factors did not alter the overall conclusions, they underscore the need for cautious interpretation of extreme effect estimates and treatment rankings.

Further studies should address the role of robotic-assisted hernia repair in elderly patients, given its potential ergonomic and technical advantages but higher cost and operative time. Long-term outcomes, including chronic pain, quality of life, late recurrence, seroma, and patient-reported recovery, should be prioritized, as these may influence patient-centered decision-making. Future multicenter trials or prospective registries with substantially larger sample sizes, ideally approaching 10,000 patients, should report age strata for octogenarians and nonagenarians, frailty status, ASA class, comorbidities, center volume, surgeon experience, discharge criteria, and 24-hour discharge rates. Understanding patient preferences is also important: some may prioritize rapid discharge, others minimizing any recurrence risk. Incorporating such preferences into future trials could refine surgical recommendations.

## Conclusion

This network meta-analysis synthesizes comparative evidence on multiple inguinal hernia repair techniques in elderly patients. The findings support the use of minimally invasive repair, particularly LR-TEP and LR-TAPP, in appropriately selected patients and in centers with suitable expertise, while identifying advanced open techniques such as PHS and plug repair as reasonable alternatives when laparoscopy is not feasible. These results should inform individualized surgical planning, with the goal of delivering safe, effective hernia repair while minimizing perioperative morbidity.

## Data Availability

The original contributions presented in the study are included in the article/Supplementary Material, further inquiries can be directed to the corresponding authors.
