# Supplementary figures and images for: Comparative efficacy and safety of surgical techniques for inguinal hernia repair in elderly patients: a network meta-analysis
Source: Front Surg. 2026 Jun 11;13:1754546. doi: 10.3389/fsurg.2026.1754546 (PMC13294185; doi:10.3389/fsurg.2026.1754546)

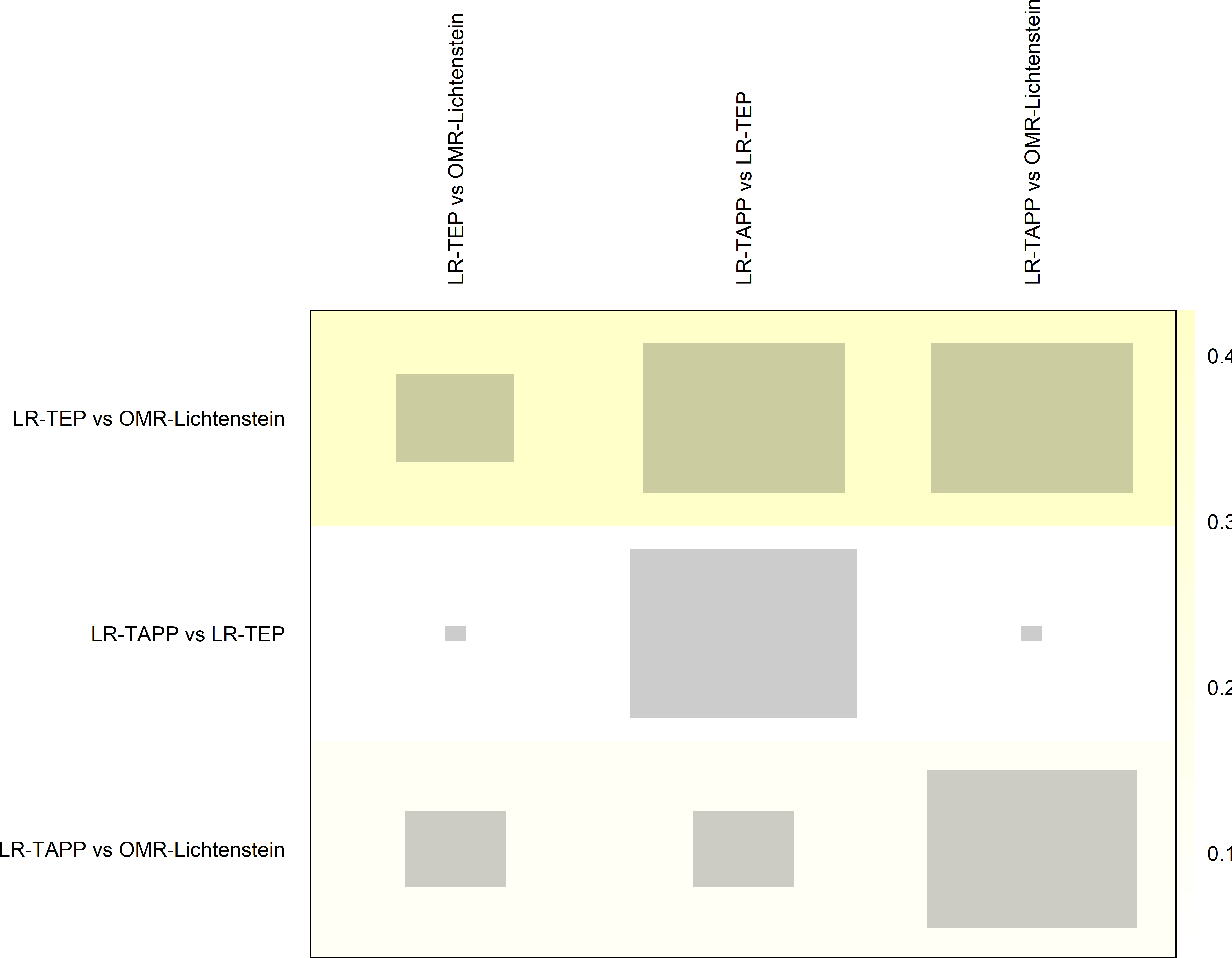

Supplement: Supplementary file 2 [file Supplementaryfile2.zip › Supplemetary Image 16.TIFF]

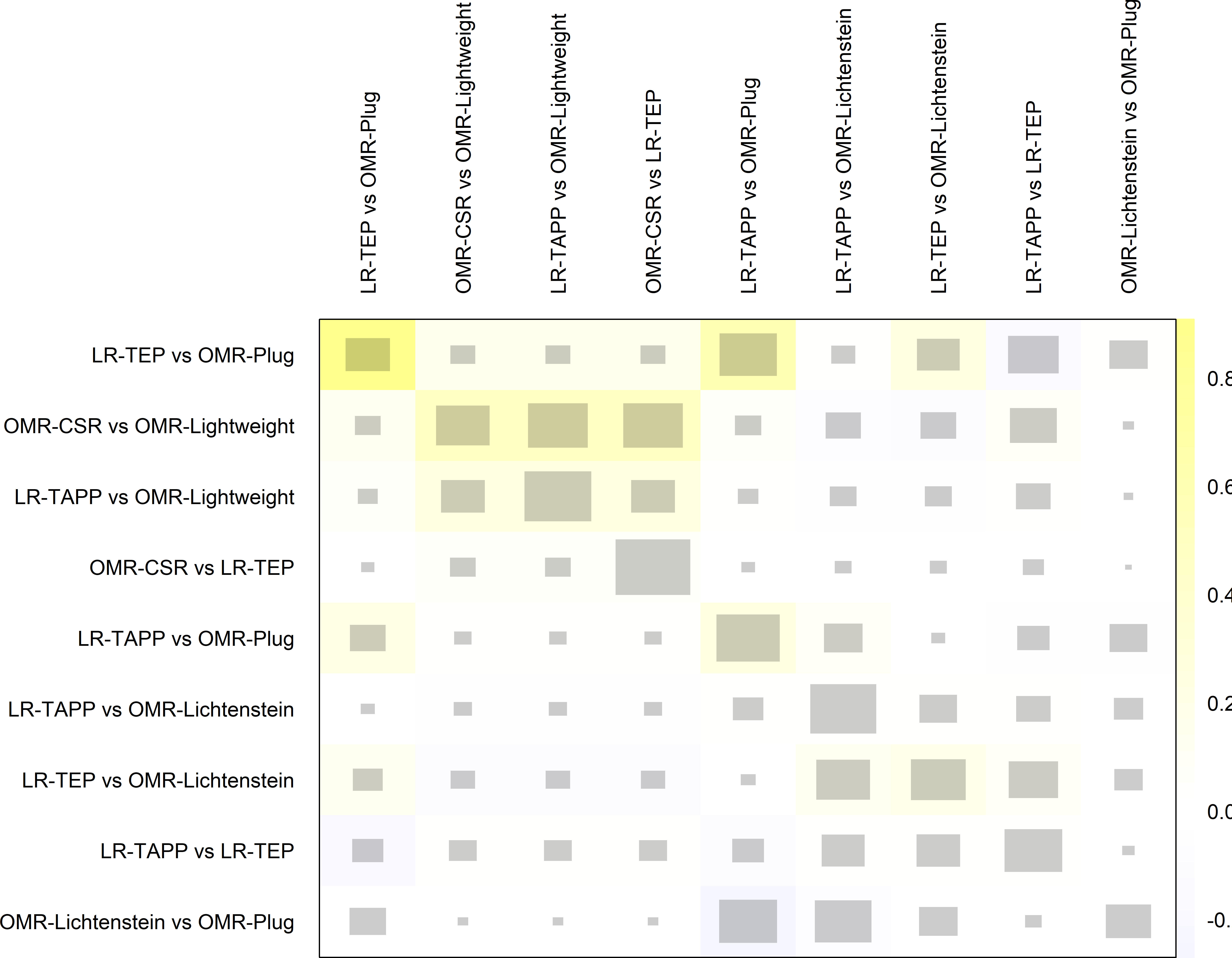

Supplement: Supplementary file 2 [file Supplementaryfile2.zip › Supplemetary Image 17.TIFF]

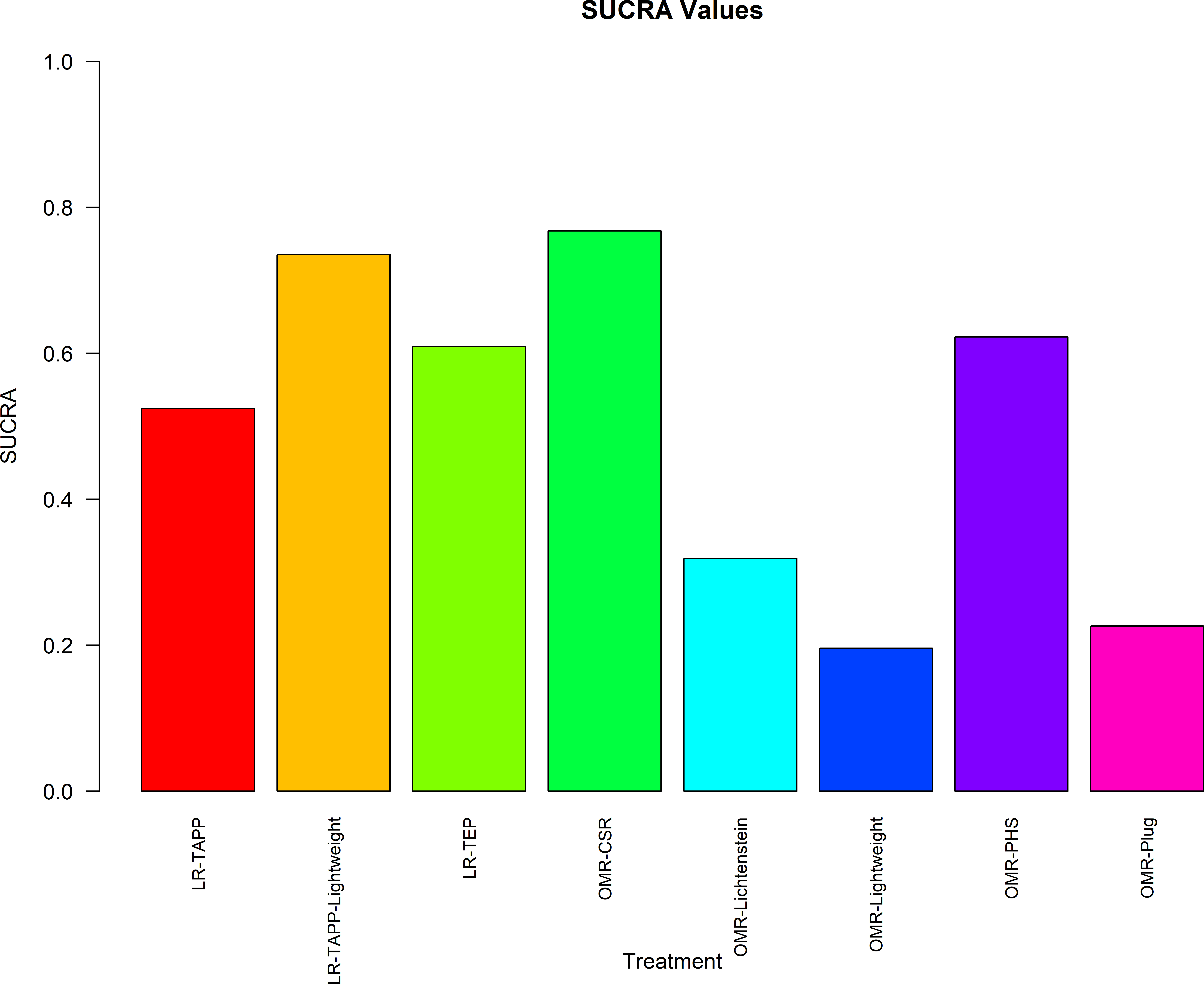

Supplement: Supplementary file 2 [file Supplementaryfile2.zip › Supplemetary Image 1.TIFF]

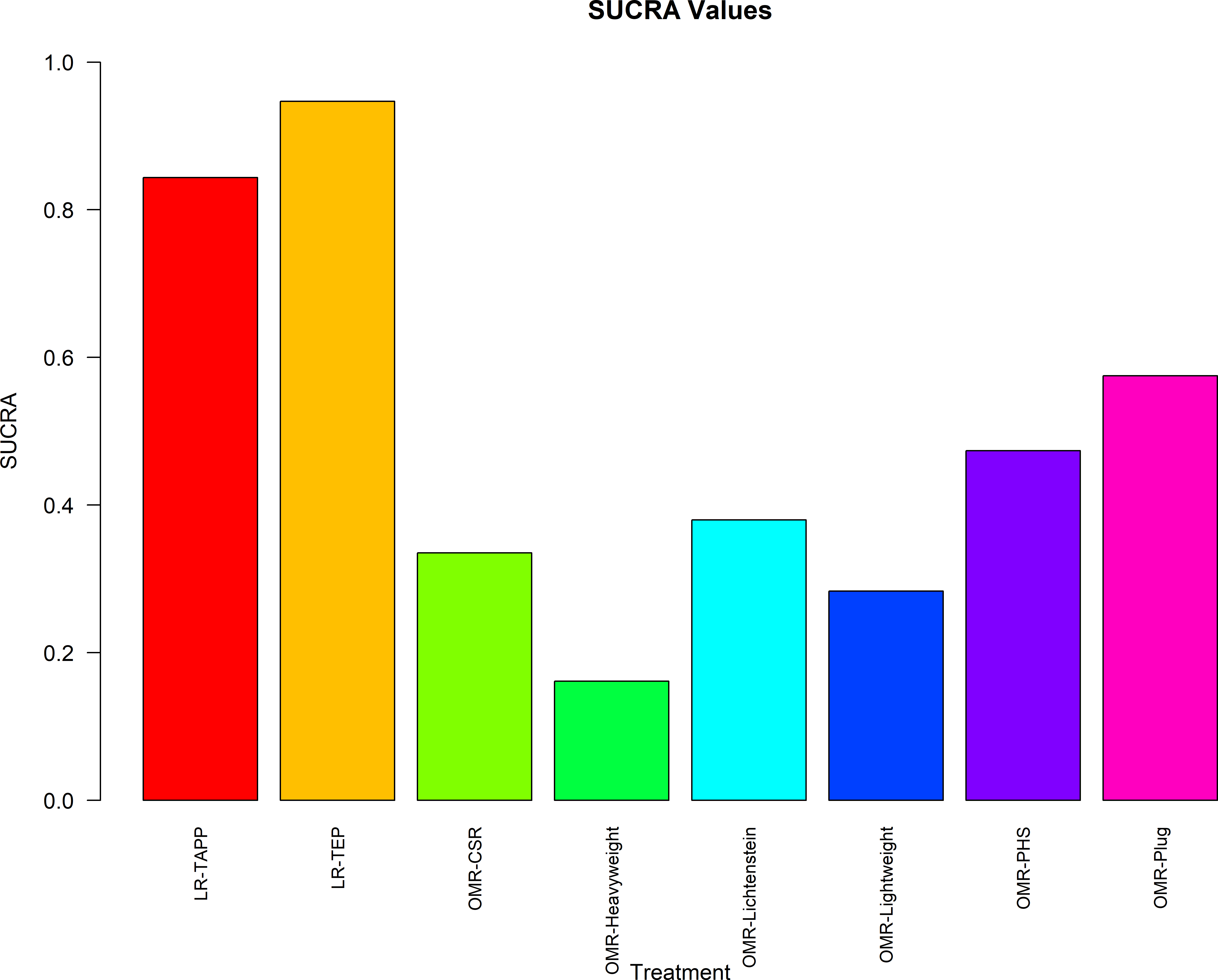

Supplement: Supplementary file 2 [file Supplementaryfile2.zip › Supplemetary Image 9.TIFF]

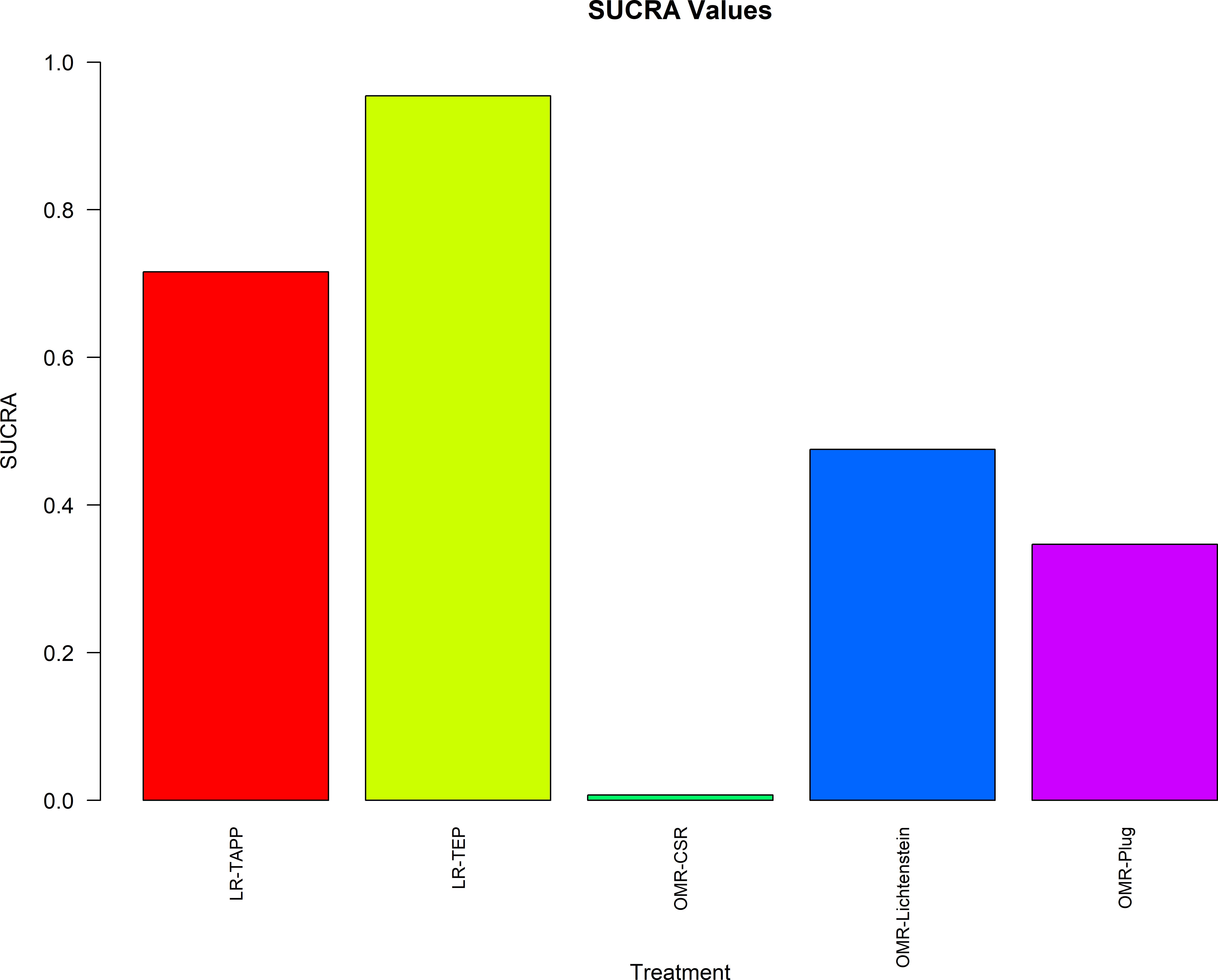

Supplement: Supplementary file 2 [file Supplementaryfile2.zip › Supplemetary Image 8.TIFF]

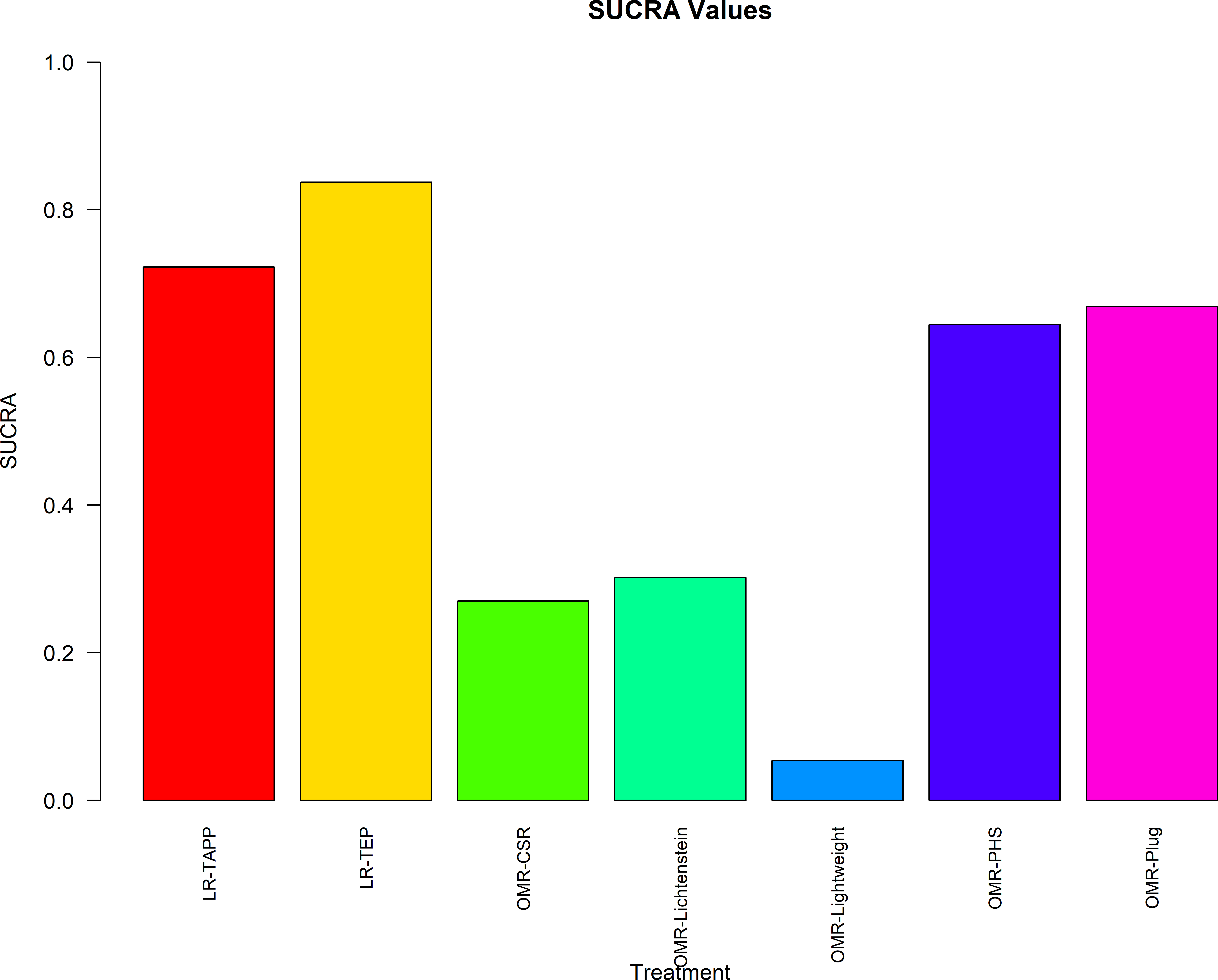

Supplement: Supplementary file 2 [file Supplementaryfile2.zip › Supplemetary Image 6.TIFF]

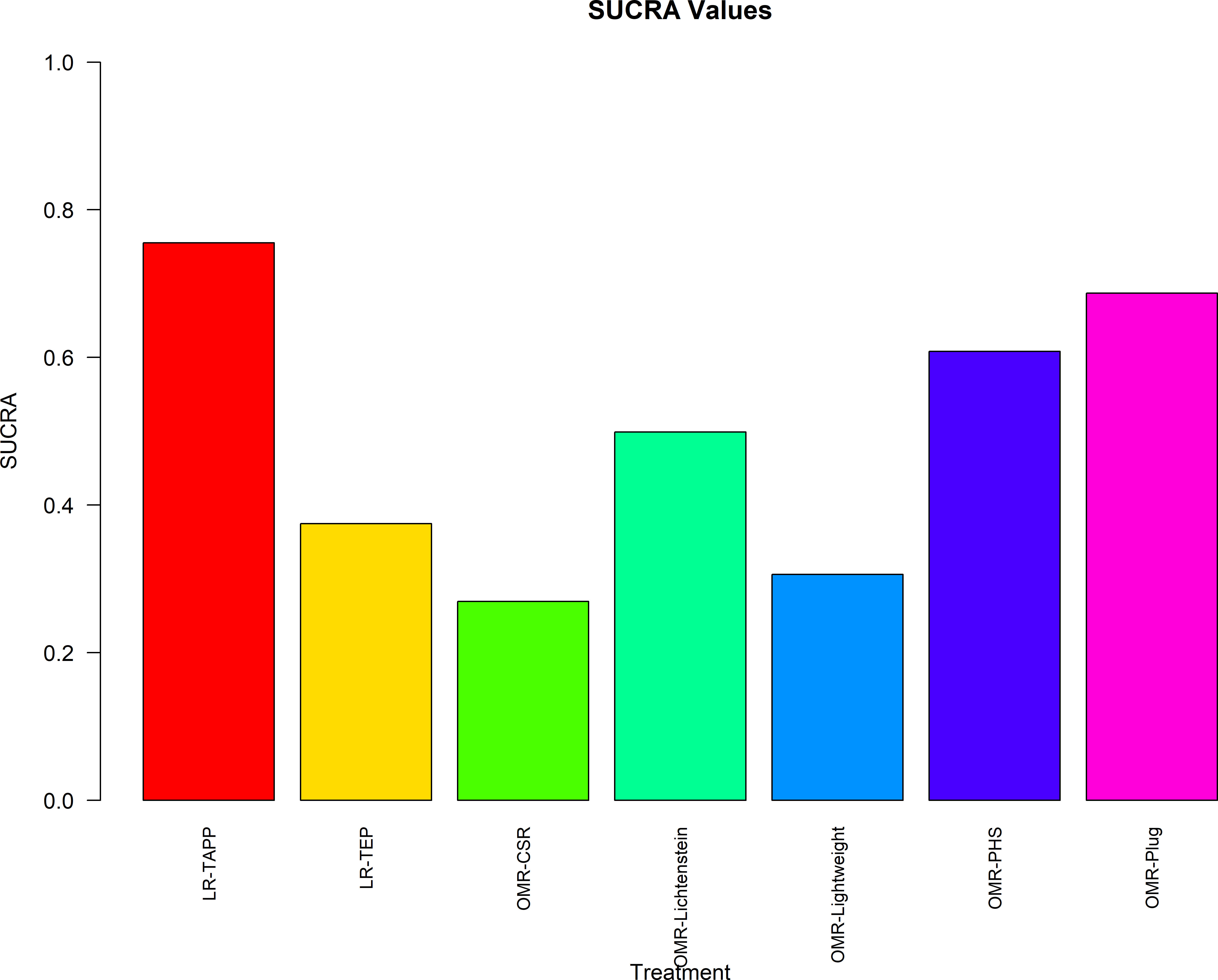

Supplement: Supplementary file 2 [file Supplementaryfile2.zip › Supplemetary Image 7.TIFF]

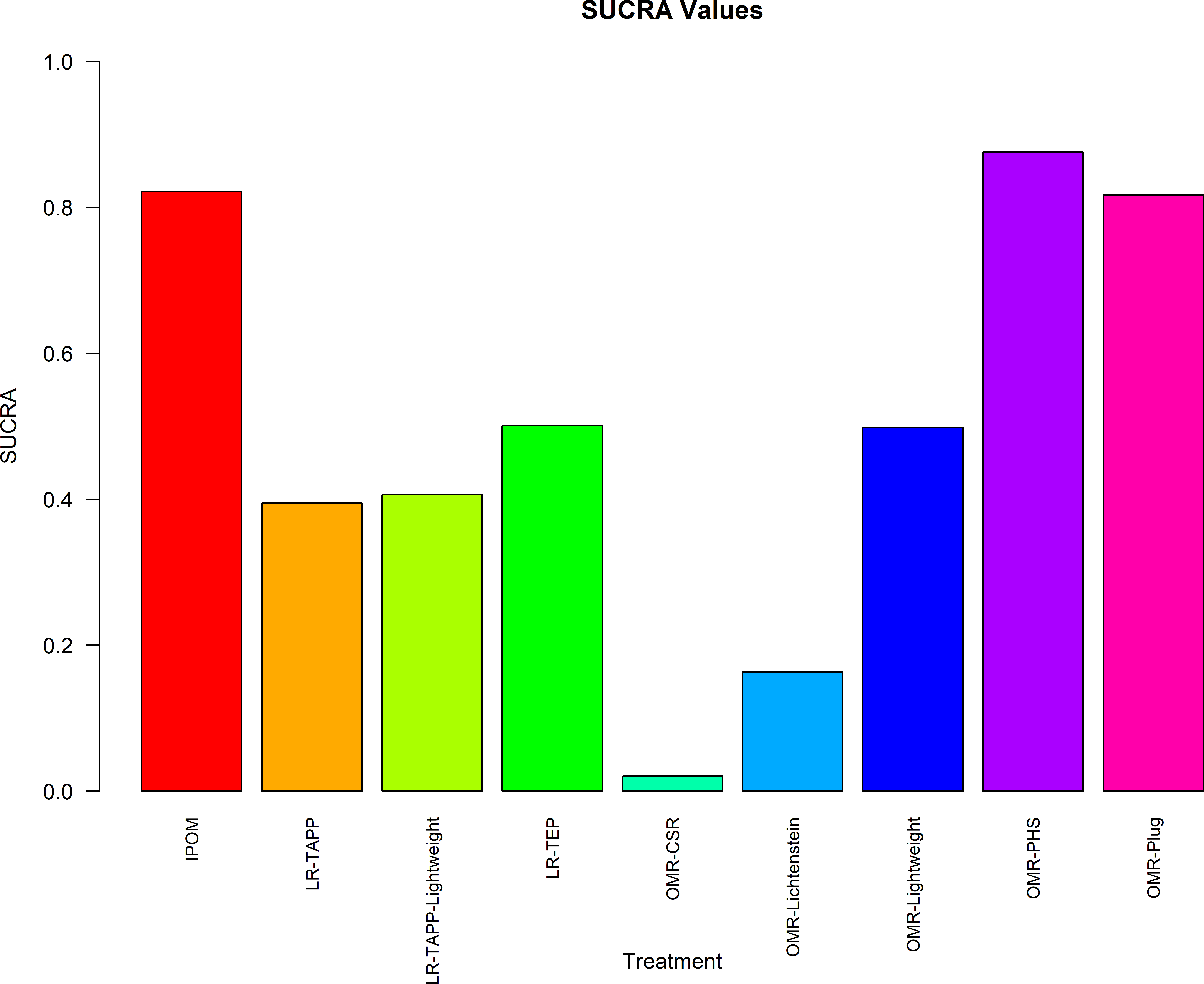

Supplement: Supplementary file 2 [file Supplementaryfile2.zip › Supplemetary Image 2.TIFF]

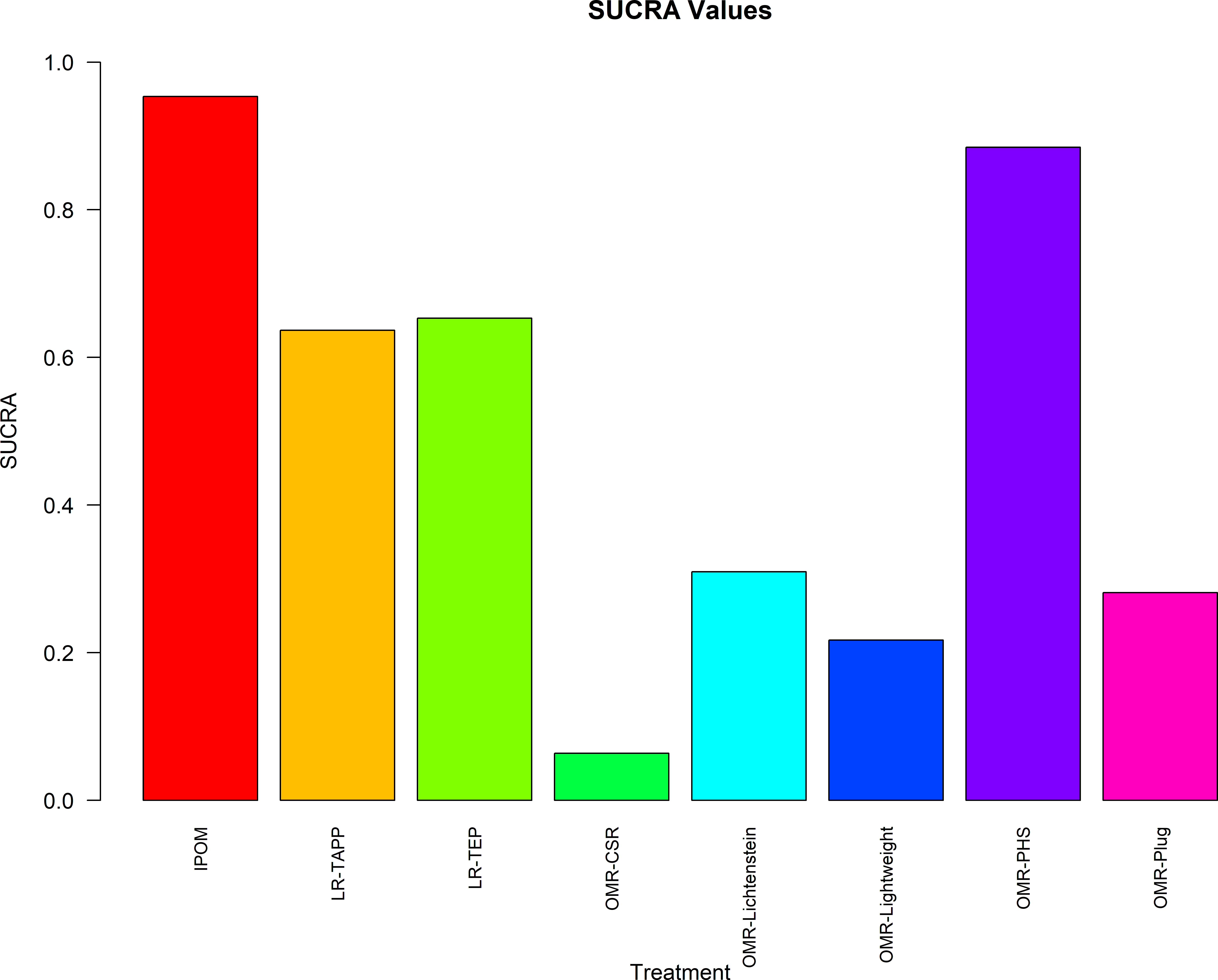

Supplement: Supplementary file 2 [file Supplementaryfile2.zip › Supplemetary Image 3.TIFF]

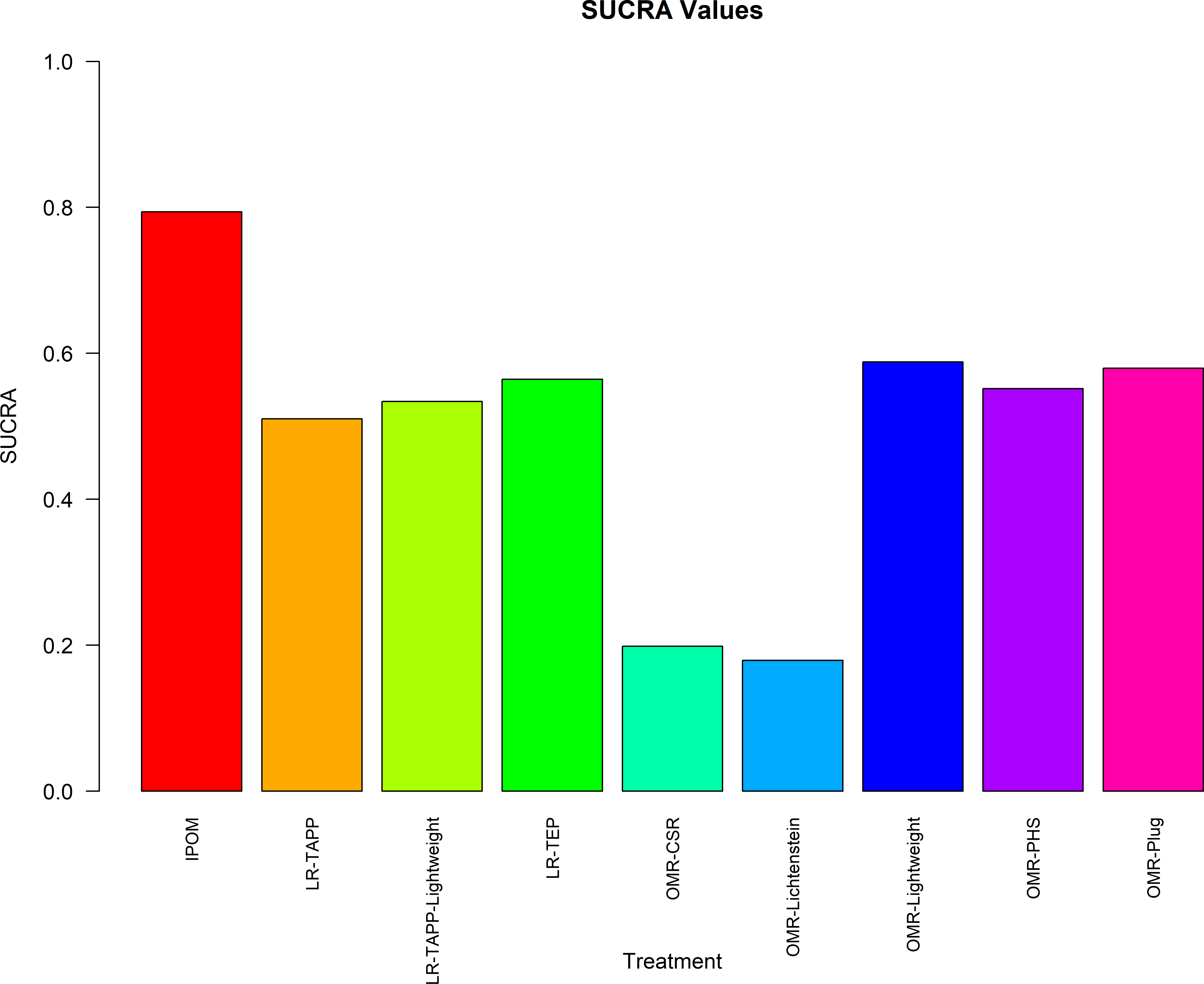

Supplement: Supplementary file 2 [file Supplementaryfile2.zip › Supplemetary Image 4.TIFF]

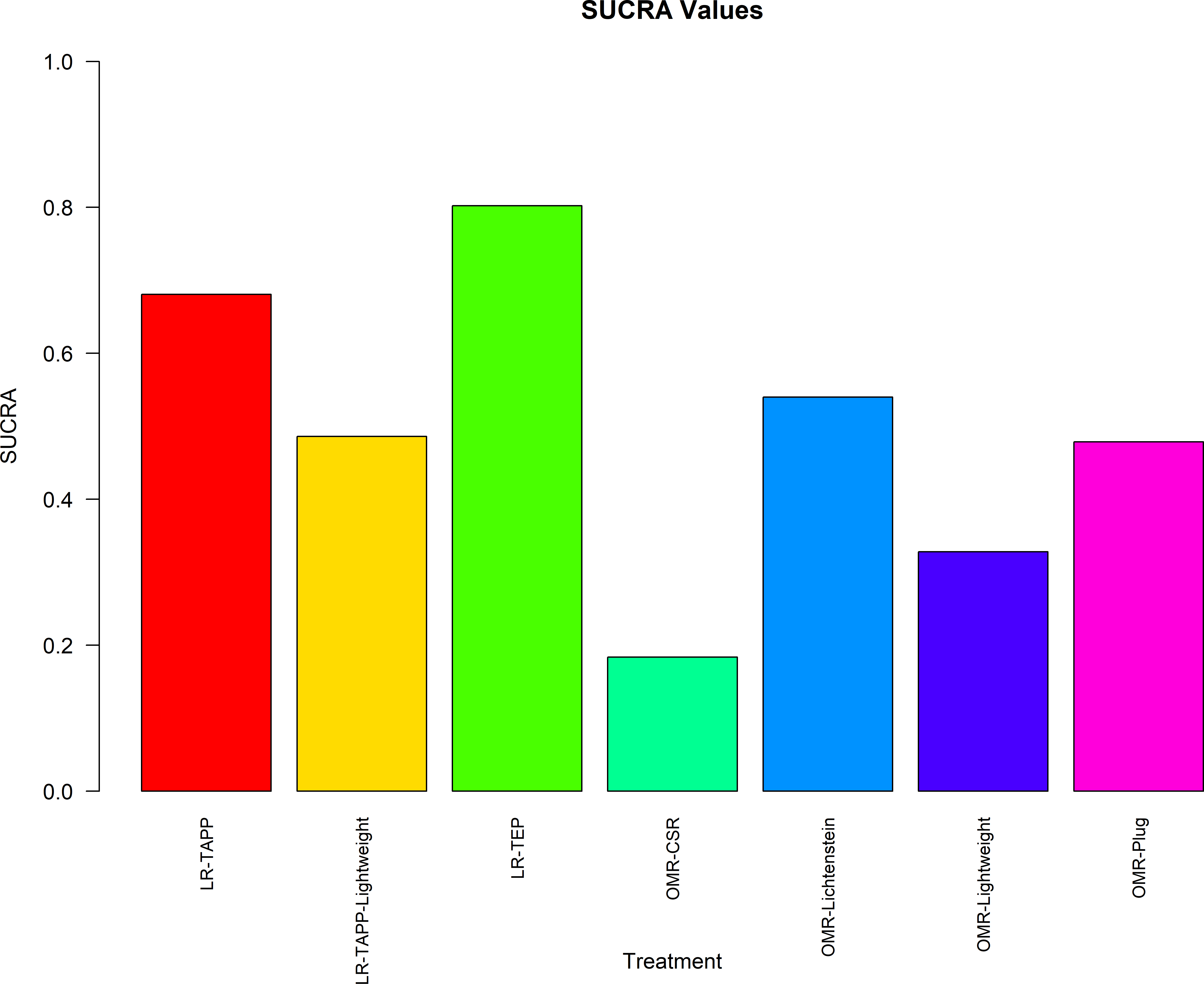

Supplement: Supplementary file 2 [file Supplementaryfile2.zip › Supplemetary Image 5.TIFF]

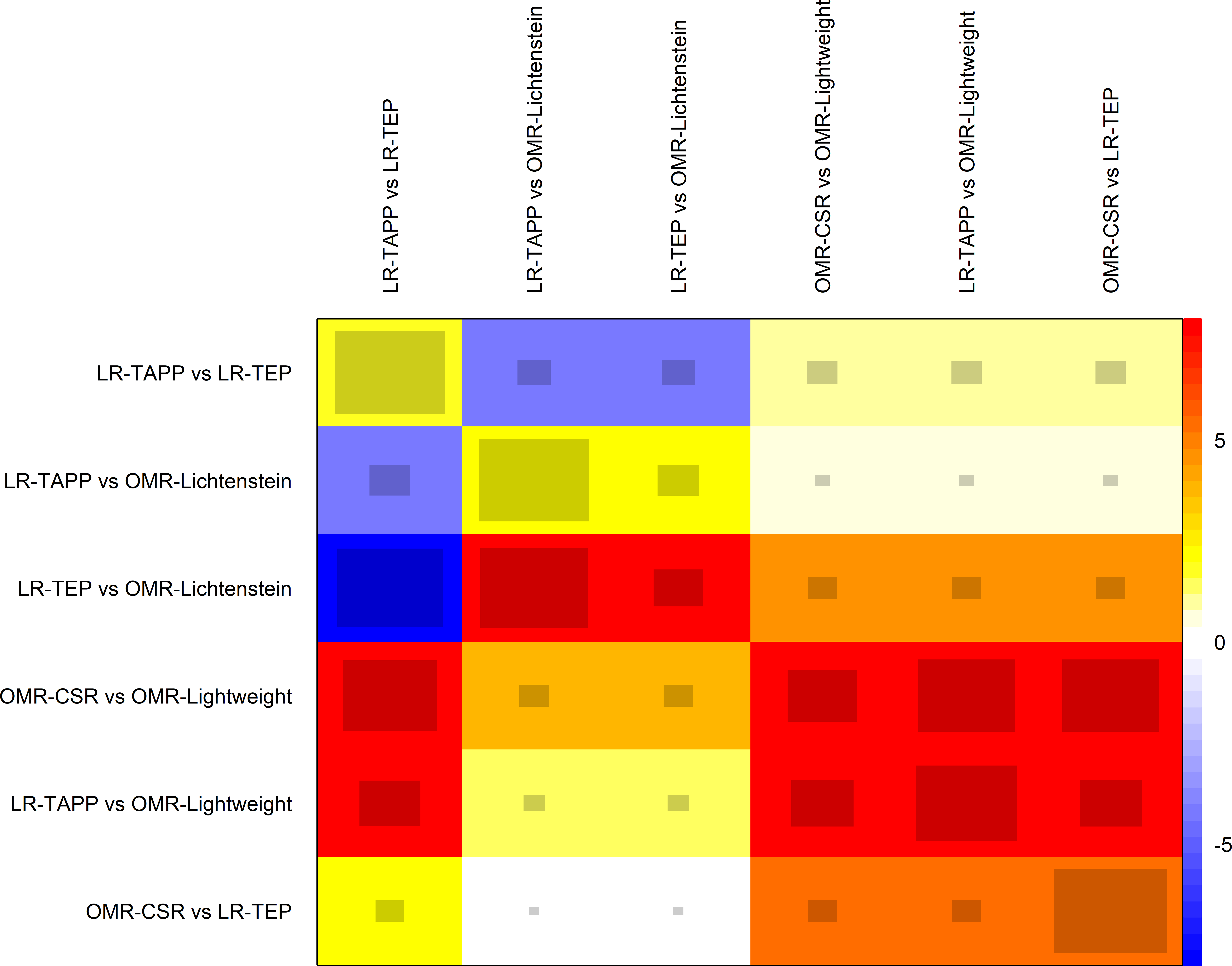

Supplement: Supplementary file 2 [file Supplementaryfile2.zip › Supplemetary Image 10.TIFF]

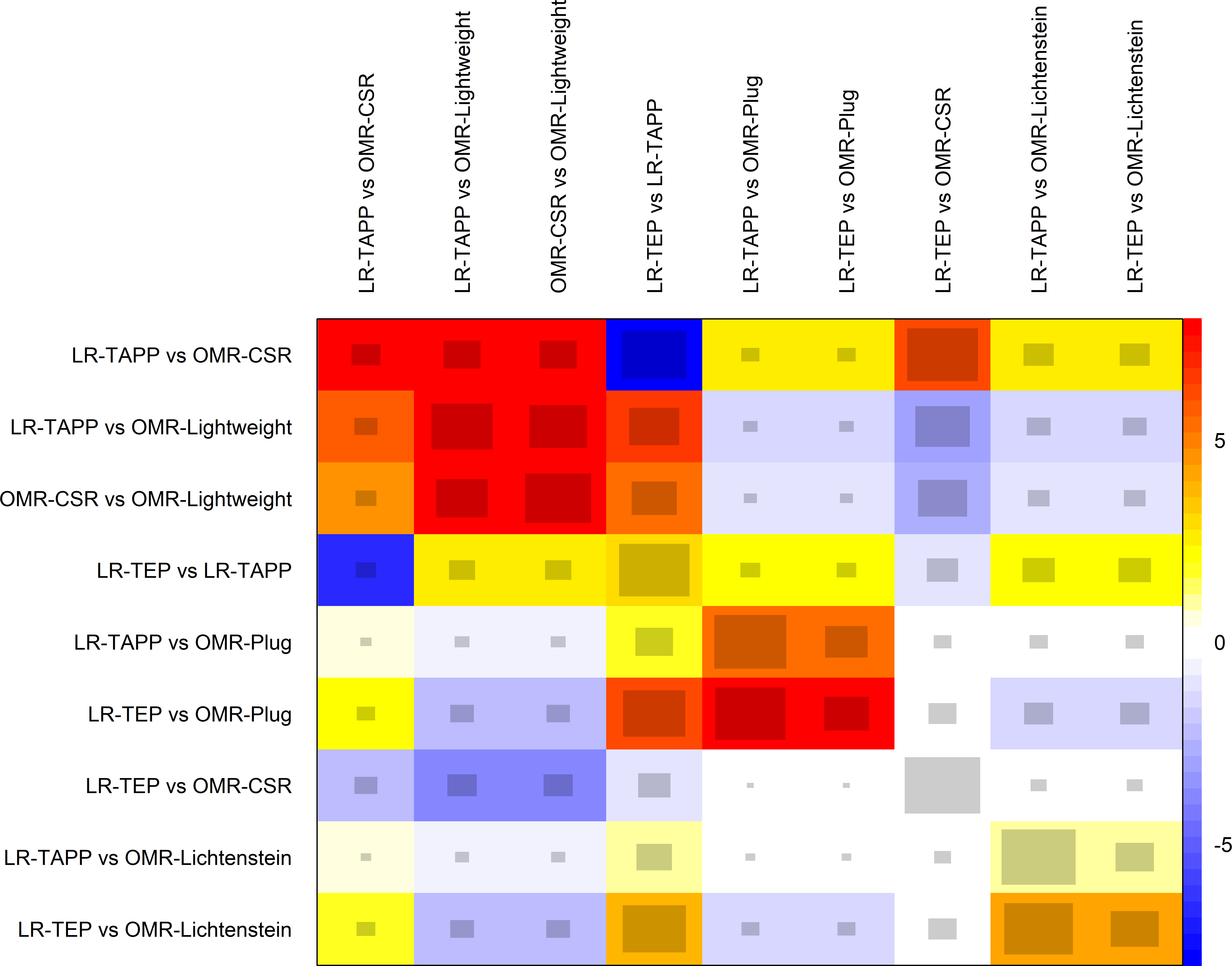

Supplement: Supplementary file 2 [file Supplementaryfile2.zip › Supplemetary Image 11.TIFF]

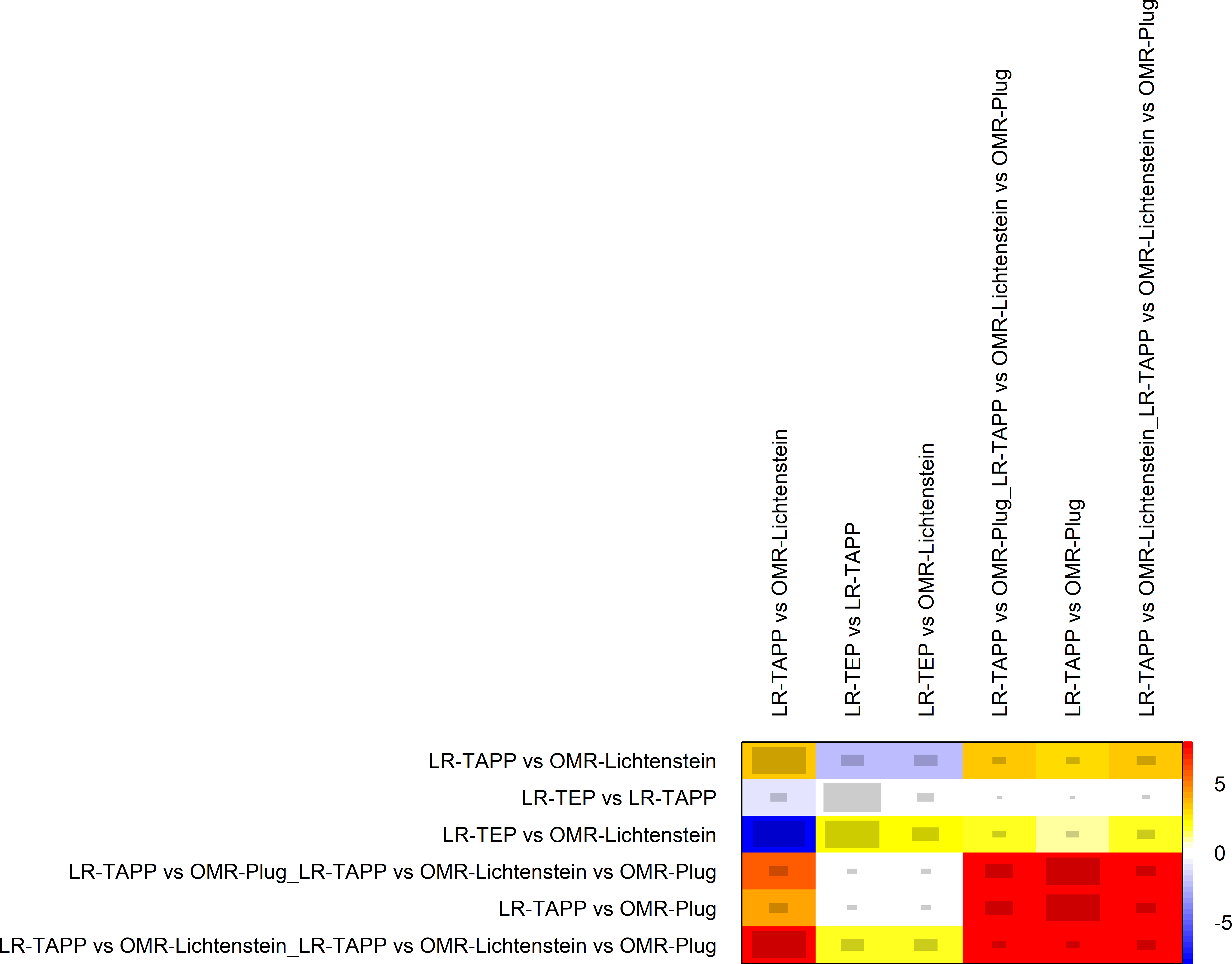

Supplement: Supplementary file 2 [file Supplementaryfile2.zip › Supplemetary Image 12.TIFF]

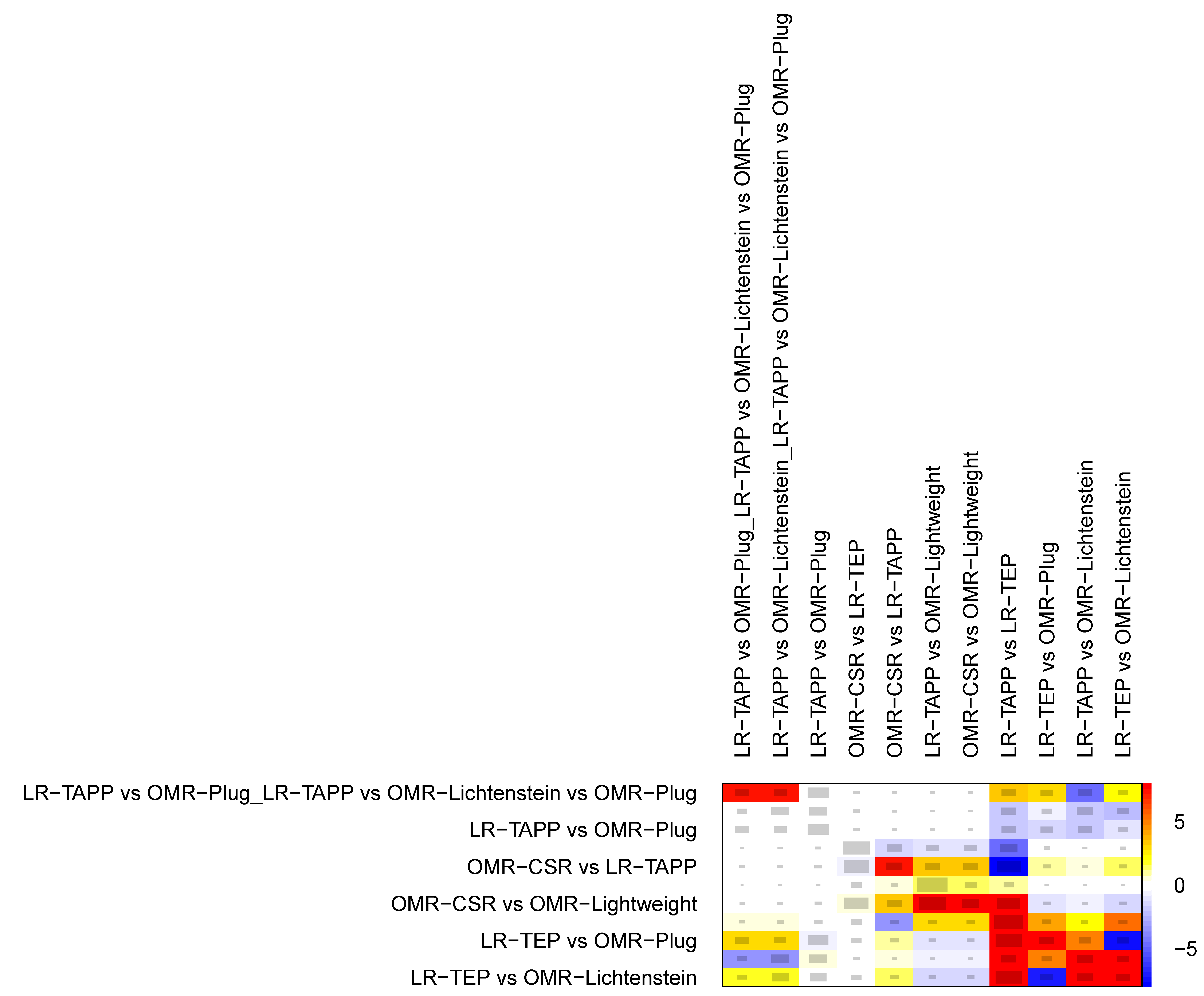

Supplement: Supplementary file 2 [file Supplementaryfile2.zip › Supplemetary Image 13.TIFF]

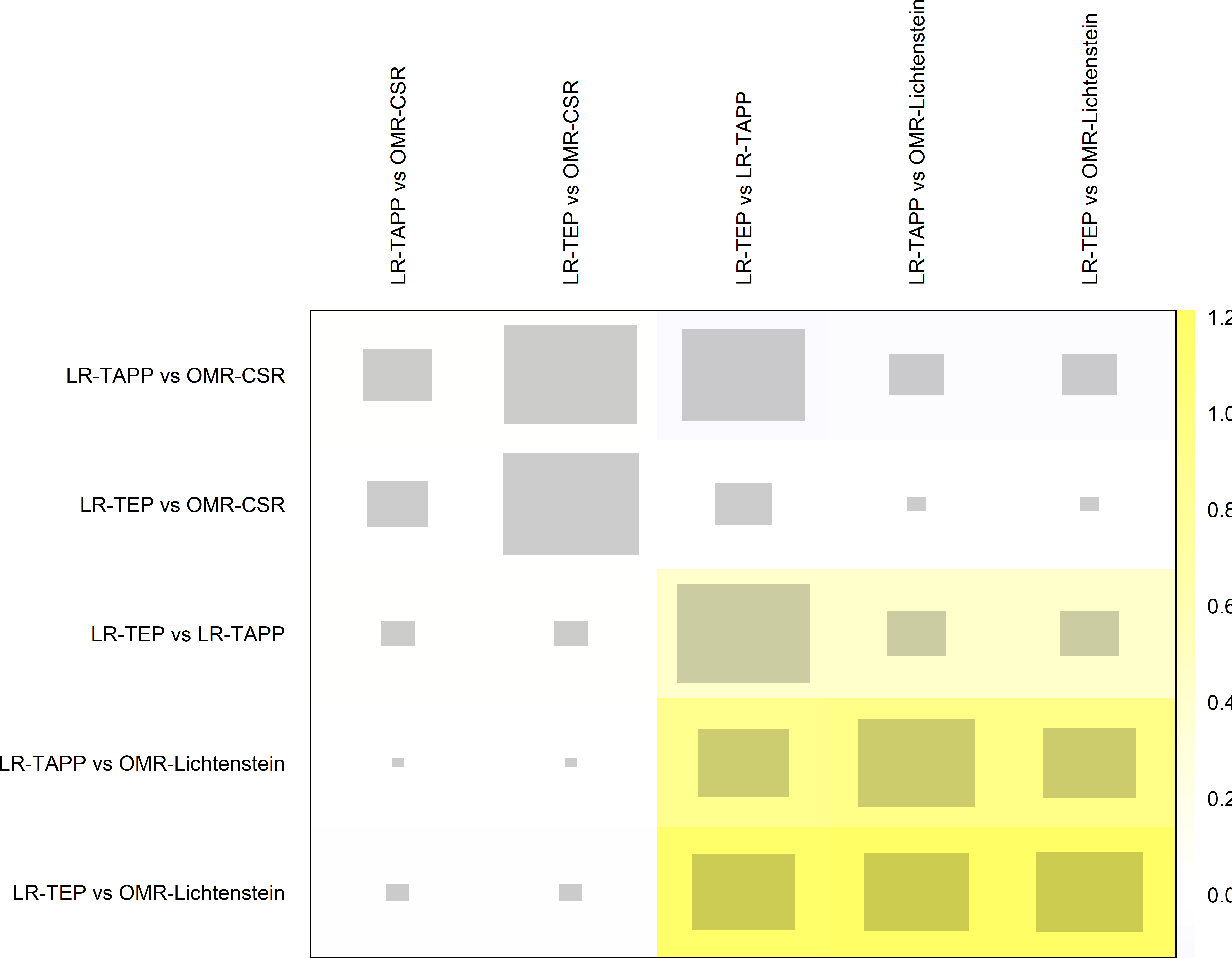

Supplement: Supplementary file 2 [file Supplementaryfile2.zip › Supplemetary Image 14.TIFF]

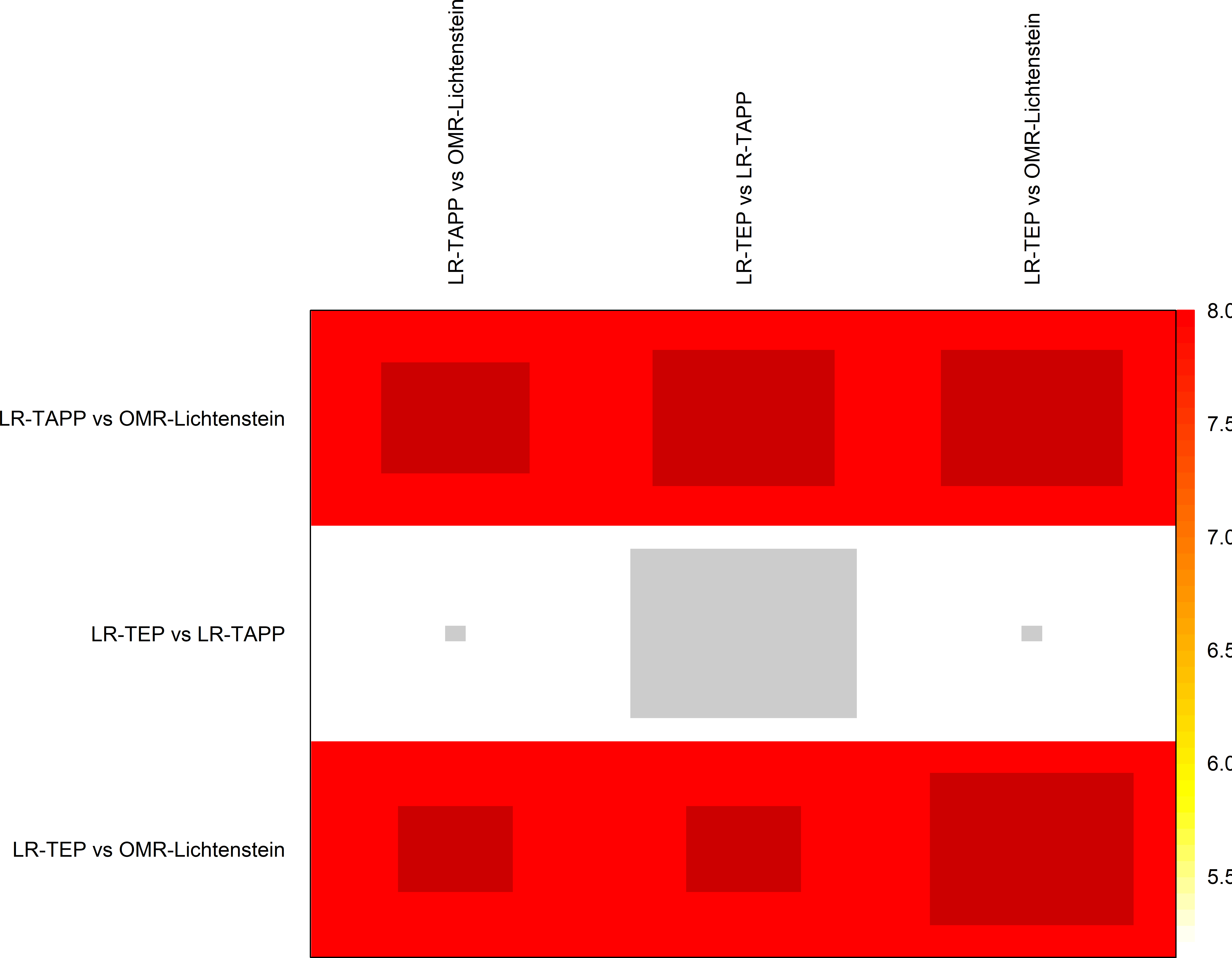

Supplement: Supplementary file 2 [file Supplementaryfile2.zip › Supplemetary Image 15.TIFF]
